# The demise of a wonder: Evolutionary history and conservation assessments of the Wonder Gecko *Teratoscincus keyserlingii* (Gekkota, Sphaerodactylidae) in Arabia

**DOI:** 10.1371/journal.pone.0244150

**Published:** 2021-01-07

**Authors:** Karin Tamar, Johannes Els, Panagiotis Kornilios, Pritpal Soorae, Pedro Tarroso, Evanthia Thanou, John Pereira, Junid Nazeer Shah, Esmat Elfaki Mohammed Elhassan, Jeruel Cabadonga Aguhob, Saoud Faisal Badaam, Mohamed Mustafa Eltayeb, Ricardo Pusey, Theodore J. Papenfuss, J. Robert Macey, Salvador Carranza

**Affiliations:** 1 Institute of Evolutionary Biology (CSIC-Universitat Pompeu Fabra), Passeig Marítim de la Barceloneta, Barcelona, Spain; 2 Environment and Protected Areas Authority, Sharjah, United Arab Emirates; 3 The Molecular Ecology Backshop, G. Lekka, Loutraki, Greece; 4 Environment Agency, Abu Dhabi, United Arab Emirates; 5 CIBIO/InBIO, Research Centre in Biodiversity and Genetic Resources, Universidade do Porto, Campus Agrário de Vairão, Rua Padre Armando Quintas, Vairão, Vila do Conde, Portugal; 6 Section of Animal Biology, Department of Biology, School of Natural Sciences, University of Patras, Patras, Greece; 7 Natural Resource Conservation Section, Environment Department, Dubai Municipality, Dubai, United Arab Emirates; 8 Museum of Vertebrate Zoology, Valley Life Sciences Building, University of California, Berkeley, CA, United States of America; 9 Peralta Genomics Institute, Chancellor’s Office, Peralta Community College District, Oakland, CA, United States of America; State Museum of Natural History, GERMANY

## Abstract

Effective biodiversity conservation planning starts with genetic characterization within and among focal populations, in order to understand the likely impact of threats for ensuring the long-term viability of a species. The Wonder Gecko, *Teratoscincus keyserlingii*, is one of nine members of the genus. This species is distributed in Iran, Afghanistan, and Pakistan, with a small isolated population in the United Arab Emirates (UAE), where it is classified nationally as Critically Endangered. Within its Arabian range, anthropogenic activity is directly linked to the species’ decline, with highly localised and severely fragmented populations. Here we describe the evolutionary history of *Teratoscincus*, by reconstructing its phylogenetic relationships and estimating its divergence times and ancestral biogeography. For conservation implications of *T*. *keyserlingii* we evaluate the genetic structure of the Arabian population using genomic data. This study supports the monophyly of most species and reveals considerable intraspecific variability in *T*. *microlepis* and *T*. *keyserlingii*, which necessitate broad systematic revisions. The UAE population of *T*. *keyserlingii* likely arrived from southern Iran during the Pleistocene and no internal structure was recovered within, implying a single population status. Regional conservation of *T*. *keyserlingii* requires improved land management and natural habitat restoration in the species’ present distribution, and expansion of current protected areas, or establishment of new areas with suitable habitat for the species, mostly in northern Abu Dhabi Emirate.

## Introduction

Arid and semi-arid regions cover almost 20% of the world’s land surface and are important for understanding global biodiversity patterns [1–4]. Biodiversity in arid regions is often perceived as poor and as less valuable than in other biogeographic regions (but see [5]), thus it is often neglected and attracts less scientific attention and conservation concern [3]. Ecosystems of arid areas, despite their wide extension and limited accessibility [6], are severely affected by anthropogenic disturbances. Threats to arid environments continue to erode local biodiversity, mostly due to urban expansion, overgrazing, desertification, agricultural expansion, drought, and armed conflicts [7–9].

The United Arab Emirates (UAE) in the Arabian Peninsula, similar to other oil producing countries of the Middle East, is developing rapidly. As a result of this extensive development, human populations are increasing, threatening wildlife populations and natural habitats [9,10]. Rapid construction and industrialization in Arabia have expanded widely, especially in recent decades, destroying and degrading unique arid habitats, particularly coastal sand dunes and salt flats (sabkhahs) [11]. Destruction or degradation of natural habitats and loss of original cover have continuously driven declines in biodiversity and ecosystem services worldwide [12,13]. Fragmentation and destruction of natural habitats through anthropogenic activity are known threats to the viability of endangered species and are recognised as important drivers to current species extinctions [14,15]. Loss of natural habitats and their fragmentation result in a landscape composed of isolated patches of suitable habitats, separated by an inhospitable matrix, and thus to the isolation of previously connected populations. This segregation leads to population size reduction, which in turn, increases the probability of extinction by demographic and/or environmental changes [16,17].

One of the most widely used indicators for assessing the health of ecosystems and their biodiversity is the conservation status of flora and fauna, which is also an important component of priority-setting exercises for species conservation. Reptiles (i.e., non-avian sauropsids) comprise a major element of the global biodiversity [18] and constitute the world’s most diverse group of terrestrial vertebrates, especially in arid regions [19]. Their population trends thus represent an indicator of a general decay of environmental health, as well as the declines of other species [20]. However, despite their relevance for many ecosystems and role as excellent models for conservation studies, knowledge or assessments for the conservation of reptiles are generally lacking, falling behind those of birds, mammals, and amphibians [19,21,22].

**Fig 1:**
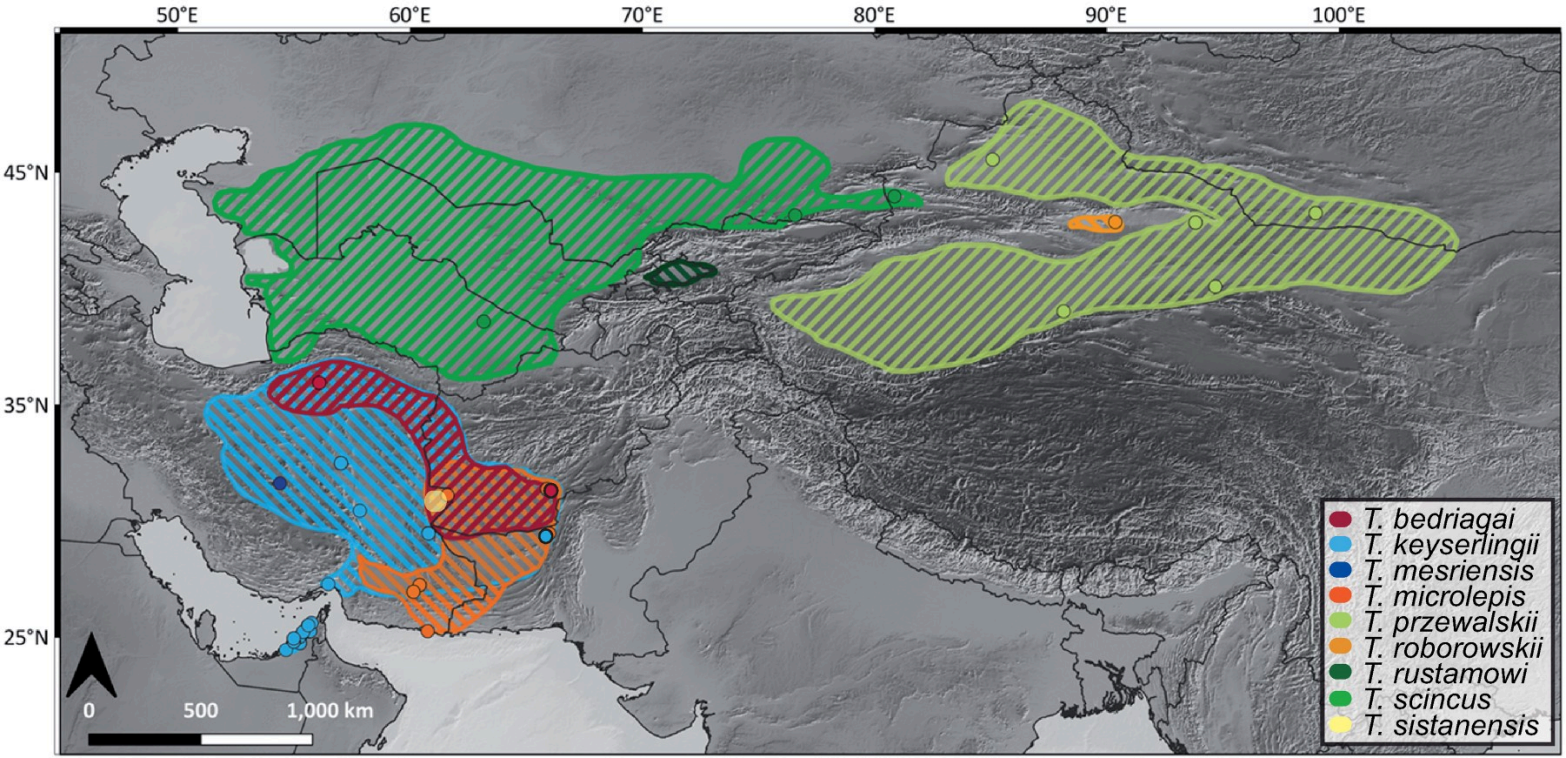
Distribution ranges of *Teratoscincus* taxa. Colours correspond to species in Figs 3 and 4. Localities of specimens sampled in this study are detailed in S1 Table. *Teratoscincus sistanensis* was not included in this study. Credits: Natural Earth contributors, SRTM.

**Fig 2:**
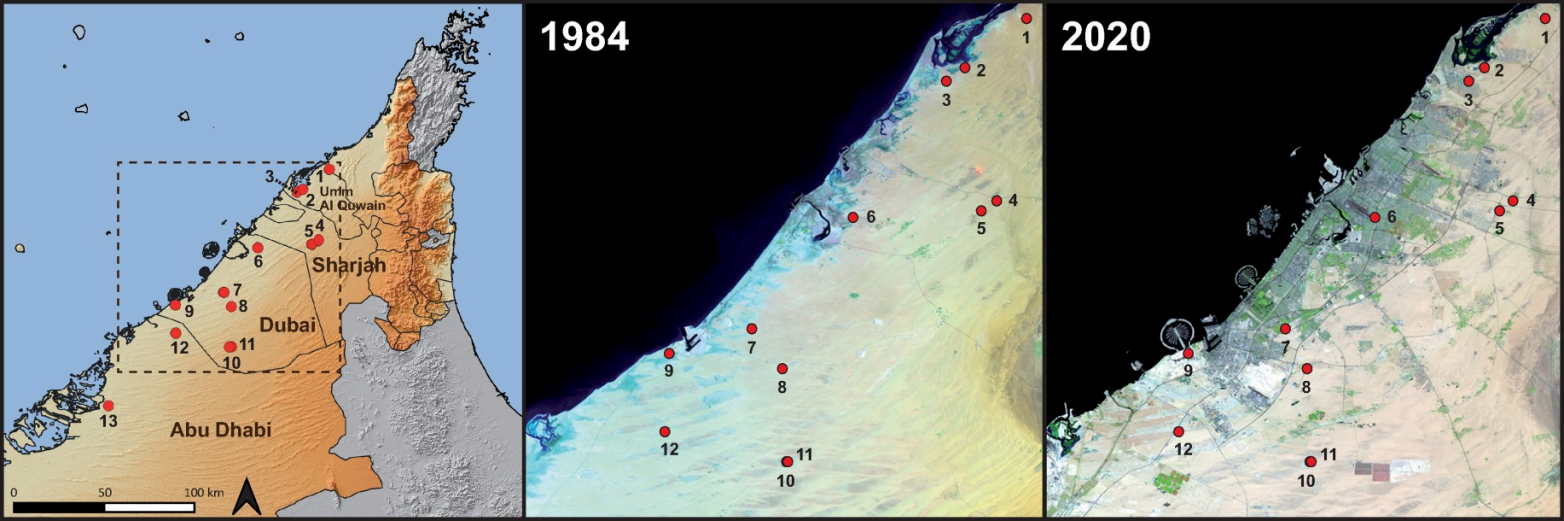
Localities of *T*. *keyserlingii* in the UAE used in this study. Images presenting the UAE coastline in the years 1984 and 2020 indicating the massive development in recent decades, which destroyed and fragmented the natural habitat of *T*. *keyserlingii*. Localities of specimens used in this study are indicated in red, with numbers corresponding to localities detailed in S1 Table. Localities 6, 7, 9, 12 are extinct due to urban development. Left map credits: OpenStreetMap contributors, SRTM. Middle and right images credits: Satellite images of environmental change, Earth Resources Observatory and Science (EROS) Center, USGS.

The drastic population decline of the Wonder Gecko, *Teratoscincus keyserlingii* Strauch, 1863, in the UAE provides particular evidence for strong anthropogenic pressures [9] and thus should be used as a model for conservation in arid regions. *Teratoscincus keyserlingii* is a member of the family Sphaerodactylidae and is one of nine recognised species of the genus *Teratoscincus*, distributed across desert regions from South-West Asia to Central Asia (Fig 1) [18]. *Teratoscincus keyserlingii* is mainly distributed in Iran, South Afghanistan and West Pakistan, with a small population in the Arabian Peninsula [18,23]. It is a psammophilous species and is often found between sand dunes, but occasionally also recorded on hard soil [24–26]. In Arabia, this species is the sole representative of the genus, being isolated from con-specific populations and con-geners by the Arabian Gulf and the Strait of Hormuz. It is locally restricted to the UAE, where it occurs over a distribution range (extent of occurrence) of less than 5,000 km^2^, with an area of occupancy of less than 100 km^2^ [27]. It occurs mostly within 30–40 km off the Gulf Coast, inhabiting sand sheets, low sand dunes, and sandy plains with vegetation below 200 m of elevation (Fig 2) [26,28]. The UAE *T*. *keyserlingii* population has been recorded since the early 1970’s [29], and currently is seldom seen being restricted to just a few localities. This population exhibits severe decline and is highly localised, divided into sub-populations, which at present are severely fragmented from each other.

Conservation status of reptiles of the Arabian Peninsula assessed by the IUCN stated that *Teratoscincus keyserlingii* was Regionally Endangered due to its restricted range in the UAE and habitat loss, owing to massive estate development [9] (see Fig 2). Recently, in the regional IUCN Red List specific assessment of *T*. *keyserlingii* in the UAE (the still unpublished conclusions of the 2018 UAE Red List of Reptiles and Amphibians, IUCN unpublished data), the species was evaluated as Critically Endangered. Its Arabian population is declining rapidly and predicted to decline by over 80–90% in the future. Major threats to the species in the UAE include habitat loss due to extensive and intensive development and urbanization, leading to severe fragmentation and isolated small populations. Minor threats include off-road driving to target animals due to local stigma, collection for international trade, and feral cats. Thus, the Arabian population experiences declines in the extent of occurrence, area of occupancy, and quality of habitat. Ongoing and planned estate development across the distribution of *T*. *keyserlingii* in the UAE may well drive the species to the brink of extinction in the Arabian Peninsula.

For effective conservation planning, genetic characterization is playing a considerable role in understanding the likely impact of threats for ensuring the long-term viability of a species [12,30]. Thus, in this study we describe the Arabian population dynamics within the framework of the genus–we generate a geographically broad phylogeny of *Teratoscincus*, with the broadest sampling to date of *T*. *keyserlingii* in the UAE. We evaluate phylogenetic structure and biogeographic history of the genus using multi-locus data and a time-calibrated Bayesian coalescent species tree. As conservation planning requires detailed genomic structure, we use genomic data and population genetics analyses to infer the *T*. *keyserlingii* Arabian population assemblage. We aim to help promote proper conservation assessments and implications that will be applied for the long-term survival of the Arabian population of *T*. *keyserlingii*.

## Materials and methods

### Ethics statement

No *in vivo* experiments were performed. Research in the UAE was conducted and specimens were sampled and manipulated, with the authorization and under strict control, and permission, of the Environmental Agencies of the three Emirates that have participated in the study: Environment and Protected Areas Authority, Government of Sharjah; Natural Resources Conservation Section, Environment Department, Dubai Municipality, Government of Dubai; Environment Agency, Government of Abu Dhabi. No animals were killed as a result of the study and all efforts were made to minimise animal suffering when obtaining a tissue sample for genetic analyses. Tissue samples from outside the UAE used for phylogenetic analyses were legally loaned from museum collections at CAS San Francisco, USA; MVZ Berkeley, USA; and KU Lawrence, USA.

This research is not institutional. As a result of its characteristics and the total control and compliance with laws, regulations, and procedures of this kind of conservation study in the UAE, this study did not need an approval by an Institutional Animal Care and Use Committee (IACUC) or ethics committee.

### Spatial data

Maps were generated using QGIS v.3.14.15 Pi [31]. The global map was extracted from the public domain Natural Earth (http://www.naturalearthdata.com) with elevation data at 1 km resolution. Spatial layers with the UAE political borders, its seven emirates and coastline were extracted from the open data initiative OpenStreetMap (https://www.openstreetmap.org). Digital elevation model (DEM), used for the elevation data, was downloaded from the reverb tool from NASA (http://reverb.echo.nasa.gov). Elevation data originated from the Shuttle Radar Topography Mission at a spatial resolution of 1 arc-second (~30 m). UAE Environmental change images were extracted from USGS Earth Resources Observatory and Science (EROS) Center [32]. Terrestrial protected areas of the UAE shapefile (S1 Appendix) was provided by the authors from the Environmental Agency from Abu Dhabi, the Environment and Protected Areas Authority, Sharjah, and the Natural Resource Conservation Section, Environment Department, Dubai Municipality.

### Taxon sampling, DNA sequencing, and alignment preparation

To evaluate phylogenetic structure within our focal species, *T*. *keyserlingii*, we gathered a dataset of the entire genus. We included a total of 123 specimens of *Teratoscincus* in our analyses, 92 newly sequenced specimens and 31 sequences retrieved from GenBank: 12 of *T*. *bedriagai*, 13 of *T*. *microlepis*, six of *T*. *roborowskii*, 12 of *T*. *przewalskii*, one of *T*. *rustamowi*, 10 of *T*. *scincus*, three of *T*. *mesriensis*, and 66 of *T*. *keyserlingii*. Monophyly of *Teratoscincus* has been supported in several studies (e.g., [33–35]), thus we used another member of the family Sphaerodactylidae, *Pristurus rupestris*, as an outgroup. Detailed information on specimens, GenBank accession numbers, and localities is presented in S1 Table.

Genomic DNA of alcohol-preserved tissue samples was extracted following standard high-salt protocols [36]. We amplified via Polymerase Chain Reaction (PCR) and bi-directionally sequenced two mitochondrial gene fragments, Cytochrome *c* oxidase subunit I (*COI*) and NADH Dehydrogenase subunit 2 (*ND2*), and two nuclear gene fragments, Melanocortin 1 receptor (*MC1R*) and Recombination activating gene 1 (*RAG1*). Primers used, their sequences, lengths of amplified regions, PCR conditions and sources are given in S2 Table. Chromatograms were checked and assembled, and the final alignments were generated using MAFFT v.7 [37] in Geneious v.9.0.5 [38]. No stop codons were detected after translation and heterozygous positions in the nuclear genes were coded according to the IUPAC ambiguity codes.

### Phylogenetic analyses

Phylogenetic relationships of *Teratoscincus* were inferred using the concatenated mtDNA-nucDNA and the *COI* datasets, performing Maximum Likelihood (ML) and Bayesian Inference (BI) analyses. The concatenated dataset was used for the general phylogenetic structure of *Teratoscincus*, and the *COI* dataset allowed broader geographic sampling and allocation of the UAE specimens to a particular *T*. *keyserlingii* lineage. Partitions and substitution models were selected using PartitionFinder v.2 [39] with the Bayesian Information Criterion (BIC) and the dataset partitioned by genes: *COI* (TrN+G), *ND2*, *RAG1* (HKY+G), and *MC1R* (HKY+I). ML analyses were performed in raxmlGUI v.1.5 [40], with the GTRGAMMA model and 100 random addition replicates. Nodal support was assessed with 1,000 bootstrap replicates. BI analyses were performed in MrBayes v.3.2.6 [41] with all parameters unlinked across partitions. Analyses were performed with four chains per run for 2 million generations, with sampling of every 200 generations and burn-in of the first 25% of posterior trees of each run. We calculated inter- and intraspecific uncorrected *p*-distance between and within *Teratoscincus* taxa for *COI* and *ND2* mitochondrial fragments, with pairwise deletion, in MEGA v.7 [42].

### Estimating a temporal framework of divergence

We estimated divergence times for *Teratoscincus* with a species-tree approach using *BEAST v.1.8.4 [43]. We assigned “species” to lineages from the phylogenetic trees described above. We calibrated the species-tree using biogeographical data from Macey et al. (1999, 2005) [44,45], supported in other studies using different calibration approaches [e.g., 34,35,46,47]: the split between *T*. *microlepis* and the remaining *Teratoscincus* species due to the rise of the Hindu Kush ~20 Mya (normal distribution; mean 21, stdev 3) and the *T*. *scincus*-*T*. *roborowskii* divergence caused by the Tien Shan–Pamir uplift ~10 Mya (normal distribution; mean 11, stdev 3; including also *T*. *przewalskii*, *T*. *keyserlingii*, and *T*. *mesriensis*). We analysed the data with the following priors (otherwise by default): *COI+ND2* and *RAG1* (HKY+G), *MC1R* (K80+G), alpha prior uniform (0–10), uncorrelated relaxed clock for the mitochondrial partition and strict clock for the nuclear loci (uniform distribution; mean 0.01 and 0.0001, respectively, 0–1). We run *BEAST three times for 200 million generations and a sampling frequency of every 20,000 generations, with 10% discarded as burn-in. Convergence, posterior trace plots, and effective sample sizes (>200) were evaluated with Tracer v.1.6 [48]. LogCombiner and TreeAnnotator were used to combine the runs and to generate the ultrametric tree.

### Ancestral range reconstruction

To infer biogeographic history and estimate ancestral ranges of *Teratoscincus*, we used a reduced dataset. Specimens selected represent lineages delimited after applying the multi-rate Poisson Tree Processes (mPTP) [49] model, and one additional specimen representing a lineage occurring in more than one discrete biogeographic area (i.e., *T*. *keyserlingii* in South-West Asia and Arabia). As the mPTP analysis relies on single locus data, we reconstructed and used a ML mitochondrial phylogenetic tree as specified above (i.e., same mitochondrial partitions and analysis parameters). Ancestral area reconstruction analysis included a reduced concatenated mtDNA-nucDNA dataset of representatives and performed using the Bayesian Stochastic Search Variable Selection (BSSVS) [50] model implemented in BEAST [51]. Partitions, models of nucleotide substitution, and priors for divergence times were as detailed above. Prior settings, MCMC chain length and sampling strategy were as in the divergence-time estimations analysis, with additional specification of symmetric discrete trait substitution model and an exponential prior for the discrete location state rate. *Teratoscincus* representatives were assigned to four discrete biogeographic areas based on their modern day distributions: (i) Central Asia (China, Mongolia); (ii) West Asia (Turkmenistan, Uzbekistan); (iii) South-West Asia (Iran, Afghanistan, Pakistan); (iv) Arabia (UAE).

### ddRAD library preparation and sequencing

We collected ddRAD data for 26 samples of *Teratoscincus keyserlingii* from the UAE (see S1 Table), following the protocol, adapters, and indices of Peterson et al. (2012) [52]. We double-digested 500 ng of genomic DNA for each sample with 20 units each of two restriction enzymes (SbfI and MspI, New England Biolabs) for 6 h at 37°C. Fragments were purified with Agencourt AMPure beads before ligation of barcoded Illumina adapters. Samples with unique adapters were pooled, and each pool of eight samples was size-selected for a distribution of fragments with a peak of 480 bp, using E-Gel SizeSelect 2% agarose gels (Thermo Fisher Scientific). Illumina multiplexing indices were ligated to individual samples using a Phusion polymerase kit (high-fidelity Taq polymerase, New England Biolabs). Final pools were sequenced on an Illumina NexSeq 500, under a 50 bp single-end read protocol at the UPF Genomics Core Facility, Barcelona, Spain.

### ddRAD bioinformatics and genome-wide SNPs

We processed raw Illumina reads using the program iPyRAD v.0.7.8 [53]. We demultiplexed samples using their unique barcode and adapter sequences. Sites with Phred quality scores under 99% (Phred score = 33) were changed into ‘N’ characters, and reads with ≥3 N’s were discarded. Within the iPyRAD pipeline, filtered reads for each sample were clustered using VSEARCH v.2.4.3 [54] and aligned with MUSCLE v.3.8.31 [55]. We assembled ddRAD data using a clustering threshold of 92%. As an additional filtering step, consensus sequences that had low coverage (<6 reads), excessive undetermined or heterozygous sites (>3) or too many haplotypes (>2) were discarded. Consensus sequences were clustered across samples using the within-sample clustering threshold (92%). Again, alignment was done with MUSCLE, applying a paralog filter that removes loci with excessive shared heterozygosity among samples (paralog filter = 200). Maximum number of SNPs per locus was set to 6. We generated final datasets that included no missing data, i.e., with all loci present for all samples. Concatenated ddRAD data generated in this study are available in S2 Appendix.

Two data-matrices were used in the analyses: the first included the complete concatenated sequence of all invariant and variable sites (“ddRAD” dataset), while the second included one random SNP from each putatively unlinked locus (“uSNPs” dataset).

### Population structure analyses and measures of genetic variation

We estimated genetic variation parameters, including percentage of polymorphic loci and percentage of polymorphic sites in the total alignment. The concatenated ddRAD dataset was used in MEGA v.7 [41] to estimate nucleotide diversity π [56] and to perform the neutrality test of Tajimaʼs D [57] for the UAE *T*. *keyserlingii* population. The R package *hierfstat* [58] was used for the estimation of basic F-statistics, specifically per locus expected and observed heterozygosity (He and Ho, respectively), overall fixation index (F_ST_) and inbreeding index (F_IS_), as well as the hybridization coefficient (F_IC_) for each studied individual. We tested whether loci departed significantly from Hardy-Weinberg (HW) equilibrium with a Fisher’s exact test and 1,000 permutations. Finally, for clusters of individuals, we estimated pairwise F_ST_ values [59] and pairwise Nei’s D genetic distances [56], which corrects for sample size.

To investigate population structure, we used the uSNPs dataset and ran a discriminant analysis of principal components (DAPC) implemented in the R package *adegenet* [60,61]. We inferred the number of clusters (max K = 10) with the *find*.*cluster* function using the Bayesian Information Criterion (BIC) and used the *optim*.*a*.*score* function to optimise the number of PCAs.

We used the same uSNPs dataset in a Bayesian clustering analysis, as implemented in STRUCTURE v.2.3.4 [62], in order to detect population structure under specified models of evolution and to define admixed individuals. Given close geographic proximity of sampling locations, we avoided the assignment of individuals into pre-defined populations, and expected some degree of gene-flow throughout the studied region. Thus, we applied a model that assumed correlated allele frequencies and admixture among clusters and tested the probability of different numbers of independent genetic clusters (K = 1–10) with 15 iterations (250.000 burn-in length and 500.000 post burn-in replicates). Distribution of allele frequencies lambda (λ) was estimated for our dataset, setting K = 1. As for prior alpha (a), specifying the degree of admixture between each sub-population, the default setting presumes clusters that are expected to contribute equally to the sample and share the same estimated value of alpha. However, unbalanced samples (different sample size per K), extensive admixture or low F_ST_ values (<0.05) can seriously affect the number of inferred Ks and the use of an alternative setting (different alpha per K, initial a<1) can better detect the true number of clusters in most cases [63]. Thus, we opted for this alternative setting with initial a = 0.3. Inferred number of population clusters was estimated by examining log-likelihood per K [lnP(X/K)] [62] and Evanno’s ΔK [64], in Clumpak online web server [65], with the ‘greedy’ option and 2,000 random input orders.

## Results

### Phylogenetic and biogeographic inferences

Our phylogenetic analyses included 123 individuals of *Teratoscincus*. The dataset totalling 2,868 bp comprised mitochondrial fragments of *COI* (n = 93, 651 bp), *ND2* (n = 98, 546 bp), and nuclear fragments of *MC1R* (n = 52, 663 bp), and *RAG1* (n = 56, 1,008 bp). Uncorrected *p*-distances of *COI* and *ND2* mitochondrial fragments between and within each species/lineage are summarised in S3 Table.

**Fig 3:**
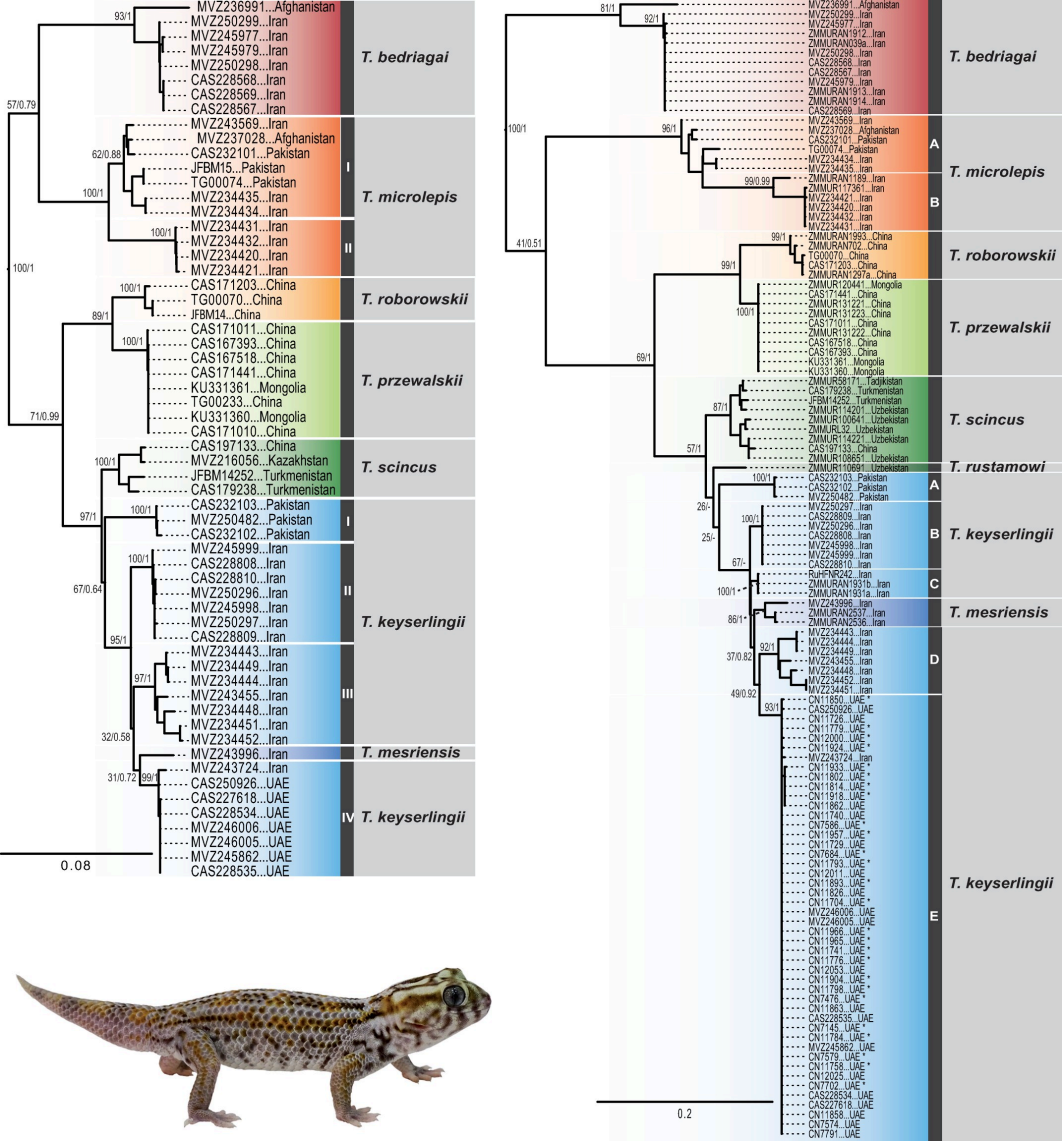
Maximum likelihood phylogenetic trees of *Teratoscincus*. (left) Phylogenetic tree using the concatenated mtDNA–nucDNA data (*COI*, *ND2*, *MC1R*, *RAG1*). (right) Phylogenetic tree using the *COI* data. Support values indicated near the nodes (ML bootstrap/Bayesian posterior probabilities). Sample codes correlate to specimens in S1 Table. Specimen depicted is *T*. *keyserlingii* from the UAE. Photo credit: Salvador Carranza.

**Fig 4:**
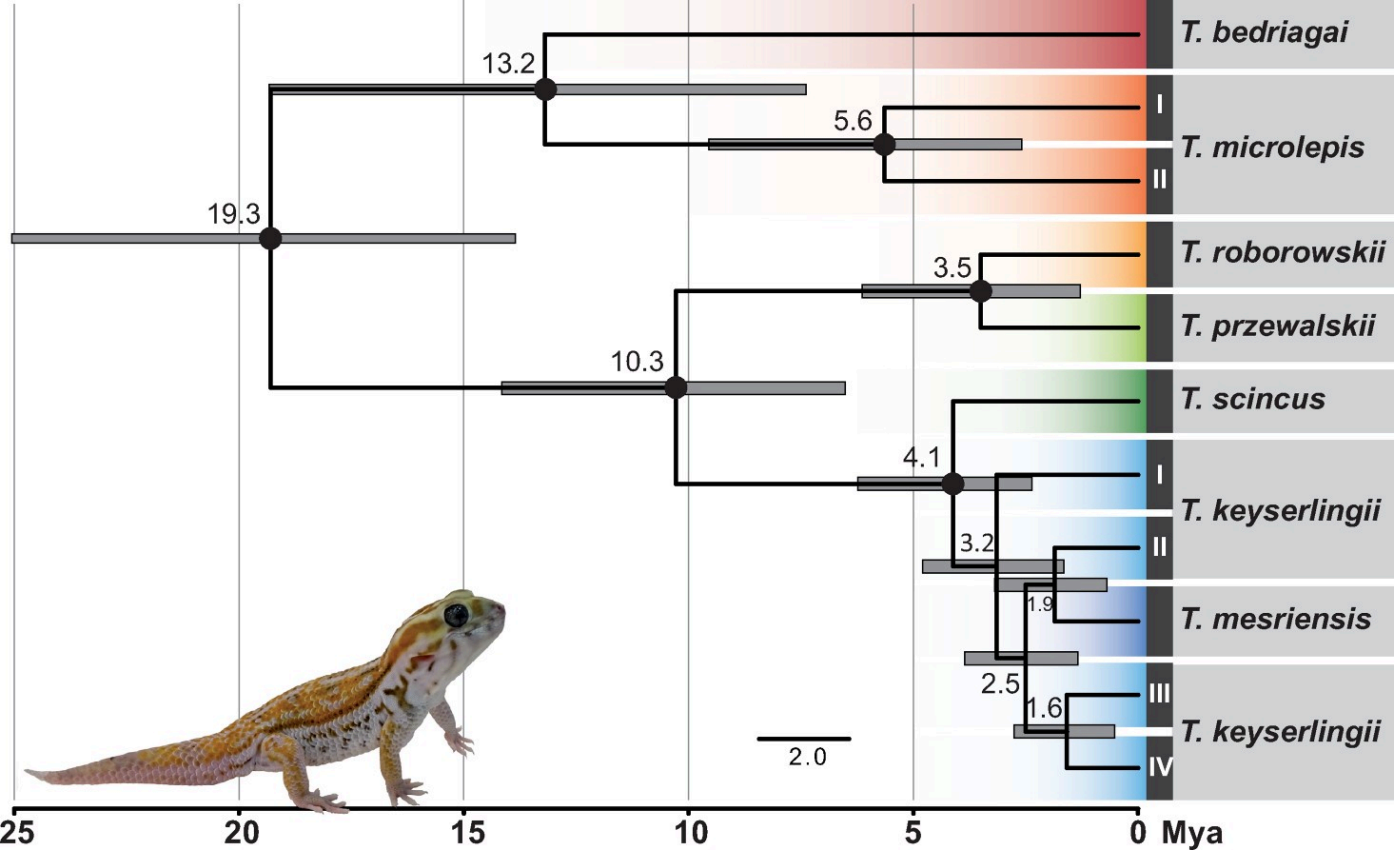
Time-calibrated species tree of *Teratoscincus*. Mean age estimates are provided above the nodes with horizontal bars representing the 95% highest posterior densities. Black circles represent nodes with posterior probability values ≥0.95. Specimen depicted is *T*. *keyserlingii* from the UAE. Photo credit: Salvador Carranza.

**Fig 5:**
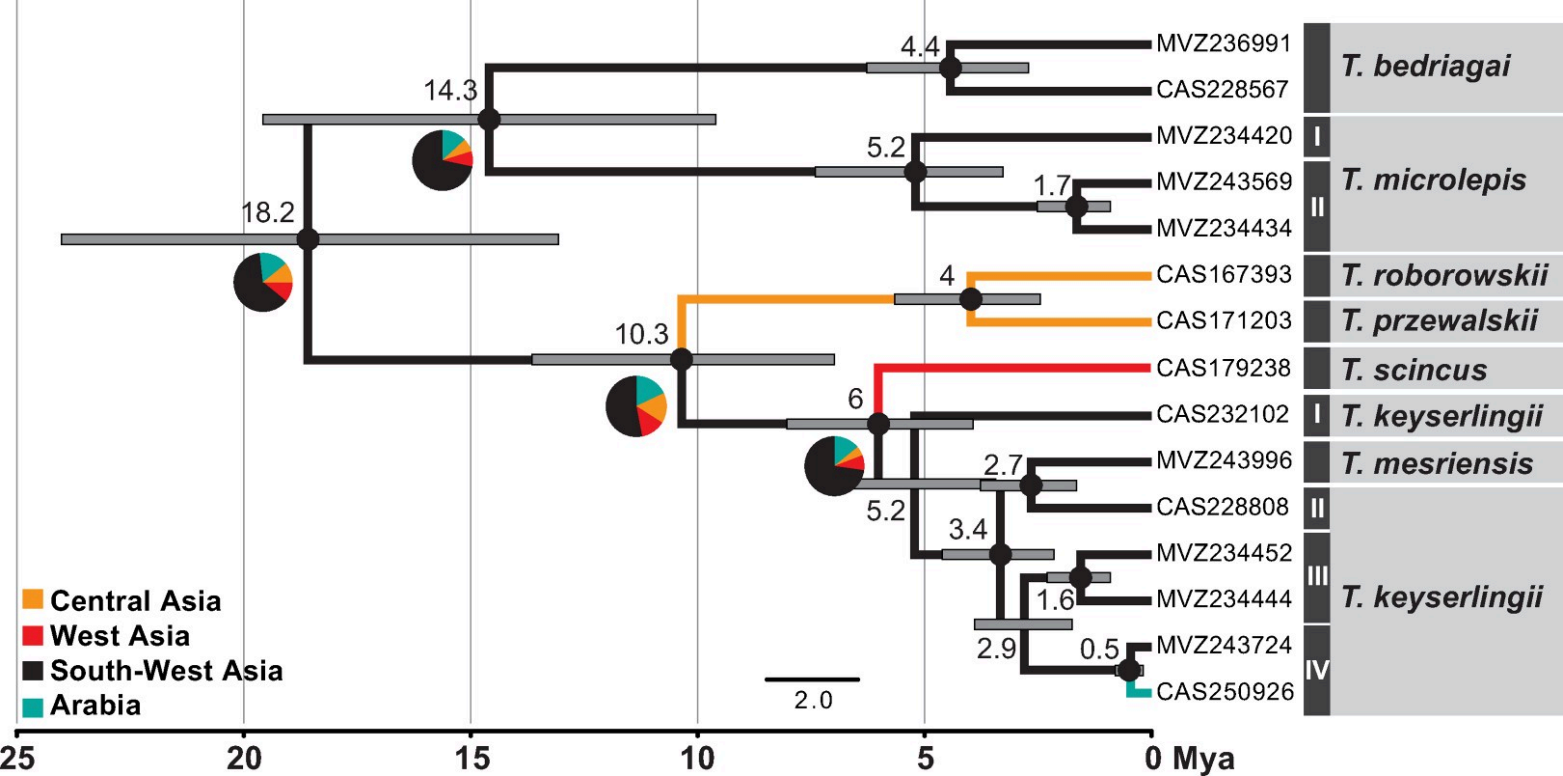
Ancestral area reconstructions of *Teratoscincus*. A Bayesian phylogenetic tree estimated using the concatenated mtDNA–nucDNA data with BSSVS. A pie chart describing the probability of each inferred area is presented near the major nodes. Branch colours indicate inferred ancestral range and the mean age estimates are provided above the nodes with horizontal bars representing the 95% highest posterior densities. Black circles represent nodes with posterior probability values ≥0.95.

The ML and BI phylogenetic trees of *Teratoscincus* using the concatenated mtDNA-nucDNA and *COI* datasets (Fig 3), the time-calibrated species tree (Fig 4), and the ancestral range reconstructions (Fig 5; mPTP tree is in S1 Fig) show a similar phylogenetic structure. *Teratoscincus* began diverging approximately 19.3 Mya (95% highest posterior density [HPD]: 13.9–25.1 Mya), most likely in South-West Asia (63% probability) with subsequent splits to north and north-west (i.e., West and Central Asia). A sister-taxa relationship between *T*. *bedriagai* and *T*. *microlepis* is recovered in the species tree and biogeographic analysis, but not supported in the concatenated mtDNA-nucDNA tree or at all recovered in the *COI* tree. *Teratoscincus microlepis* shows high intraspecific diversity and is divided into two distinct lineages, which split around 5.6 Mya (95% HPD: 2.6–9.6 Mya) and have a genetic distance of 8.3% in *COI* and 8.5% in *ND2* (S3 Table). A sister taxon relationship between *T*. *roborowskii* and *T*. *przewalskii* was supported in all trees, diverging ca. 3.5 Mya (95% HPD: 1.3–6.1 Mya). The remaining species comprise a well-supported clade diverging approximately 4 Mya (95% HPD: 2.4–6.2 Mya), yet relationships among them are not supported in all trees.

*Teratoscincus keyserlingii* is paraphyletic with respect to *T*. *mesriensis*, and shows great intraspecific diversity, with four (I–IV) and five (A–E) distinct lineages in the concatenated mtDNA-nucDNA and *COI* datasets, respectively. These lineages comprise specimens from Pakistan (lineage I/A), Iran (lineages II/B, C, III/D), and Iran and the UAE (lineage IV/E). Genetic distance among these lineages of *T*. *keyserlingii* ranged between 3.4–9.3% in *COI* and 5.1–9.4% in *ND2*. Lineage IV/E of *T*. *keyserlingii* comprised all the specimens sampled from across the UAE. These specimens clustered together with one specimen from southern Iran collected near the city of Bandar Abbas on the shores of the Strait of Hormuz (Fig 1 and S1 Table). Divergence within this lineage, between southern Iran and the UAE was recovered in our analyses at 0.5 Mya.

### Genome-wide data

For the 26 specimens of *T*. *keyserlingii* from the UAE, we obtained more than 70 million reads, with the number of reads per individual ranging from 1.17 to 4.03 million (mean 2.71). The total number of pre-filtered loci was 34,487, which dropped to 33,123 after applying all quality control filters, and to 3,912 in the final dataset, after applying a conservative filter of “all loci present in all samples”. Most loci were found to be monomorphic (3,542), while 282 included one SNP, 56 had two SNPs, 28 had three SNPs and only four loci were found to include the maximum observed number of four polymorphisms. Consequently, the final concatenated ddRAD dataset was 136,177 bp long and included a total of 494 SNPs and 370 unlinked SNPs (uSNPs).

### Population structure and genetic variation within *T*. *keyserlingii* from the UAE

Percentages of polymorphic loci and polymorphic sites in our concatenated ddRAD dataset were approximately 0.095 and 0.004, respectively, and the estimated π value was 1.28x10^-4^. Tajima’s D test of neutrality resulted in a value of -2.233. Basic F-statistics estimated from the uSNPs dataset showed a significant departure from the HW equilibrium in less than 5% of the loci (p<0.05), but an excess of homozygosity in most of them. Average F_ST_ and F_IS_ values over all loci were 0.19 and -0.18, respectively. The individual inbreeding coefficient (F_IC_) ranged between 0.44–0.52.

The optimal number of clusters obtained from DAPC was K = 1 (S2 Fig). STRUCTURE analysis recovered a slightly higher possibility for K = 3 (mean LnProb = -6,275 and mean similarity score = 0.926 for all 15 iterations) than K = 1 (mean LnProb = -6,403) (S3 Fig). This result persisted under both alternative settings of the alpha prior and even when different evolutionary models were tested (uncorrelated frequencies or no-admixture model). Most individuals were grouped in one cluster widely distributed in all sampling locations. Each of the other two presumed clusters grouped two individuals; in each case these were specimens sampled in the same exact location or in locations less than 300 m apart. Individuals belonging to the widespread cluster were also captured in the same locations, while some admixture was found between the widespread and the two local clusters (S3 Fig). Genetic differentiation among clusters was high, with Nei’s D ranging from 0.15 between the widespread cluster and each of the two local ones to 0.47 between the two local clusters. Pairwise F_ST_ values were similarly high (0.11–0.53).

## Discussion

The coupled phylogenetic-genomic approach carried out in this study for *Teratoscincus* accomplishes the task of combining broad taxonomic and geographic sampling for time-calibrated species tree inference to investigate the origin and evolution of the genus, together with including valuable information gained from genomic data for the population structure of *T*. *keyserlingii* in the UAE.

### Evolutionary history of *Teratoscincus*

Our emphasis on geographic range-wide diversification patterns enabled us to estimate relationships among almost all described *Teratoscincus* species and identify natural groupings within taxa. Congruent phylogenetic structure and monophyletic groups of species were recovered in previous studies of *Teratoscincus* [44,45,66], supported by studies incorporating other taxa (e.g., [33–35,46,67]), and corroborated by our study. We recovered monophyly of most recognised species, along with high genetic and geographic diversity within *T*. *microlepis* and our non-monophyletic focal species, *T*. *keyserlingii*. Sister-species topology is mainly supported in the species tree and the biogeographic reconstruction, though with only weak or no support in the concatenated mtDNA-nucDNA and *COI* trees. This is especially evident in geographically proximate species, and at least in one sympatric location, of *T*. *microlepis* and *T*. *bedriagai*. Divergence time estimations using the concatenated mtDNA-nucDNA dataset were generally similar though older, as expected, from the species tree.

Geographic limits of the major phylogenetic groupings of *Teratoscincus* correspond to mountain ranges uplifting in Asia caused by the Indo-Eurasian and Arabian-Eurasian tectonic collisions (Fig 1). These continuous ridges likely created geographic barriers restricting past and current species’ distributions, promoting vicariance, separating the genus into biogeographical regions. Macey et al. (1999, 2005) [44,45], having representatives of major phylogenetic groupings within *Teratoscincus*, previously hypothesised tectonic scenarios responsible for key divergences within the genus. Topology and timing of the proposed orogenic events have been supported in later studies (e.g., [34,35,46,67]), and the broader geographic and taxonomic sampling in this study further strengthen their assessments. Divergence within *Teratoscincus* began in the early Miocene and likely originated in South-West Asia. An ancestral population probably diverged due to tectonic uplifts of the Hindu Kush and Karakorum mountain ranges in northern Afghanistan and Kashmir [68–70]. This event divided *Teratoscincus* into two–a southern group of *T*. *microlepis* and *T*. *bedriagai* in the general region of Afghanistan-Iran, and a northern one, the ancestral population of the remaining species. Rise of the Tien Shan and Pamir mountain ranges in western China and eastern Tajikistan during the middle Miocene [71–73] likely promoted an east-west divergence of remaining species–an eastern population dispersed into western China and southern Mongolia, and a western population spread to the south and east of the Caspian Basin. The eastern population diverged into two recognised species, *T*. *roborowskii* and *T*. *przewalskii*, after the rise of the Kuruk Tagh mountain range (an eastern extension of the Tien Shan range) in the middle Pliocene [74–76], restricting *T*. *roborowskii* to the Turpan Basin in China. The western population diverged into northern *T*. *scincus* and southern *T*. *keyserlingii*, likely as a result of the Kopet-Dagh mountain uplifting along the Iran-Turkmenistan border in the early Pliocene [77,78].

Taxonomic concerns are raised regarding *T*. *microlepis* and *T*. *keyserlingii* due to considerable intraspecific diversity within them. *Teratoscincus microlepis* presents a late Miocene diversification, with one lineage (lineage II/B) restricted to the Hamun-e Jaz Murian Basin in southeast Iran, where it is sympatric with *T*. *keyserlingii*, spanning across the southern provinces of Kerman and Sistan and Baluchistan. A second lineage (lineage I/A) is widespread in eastern and southern Iran, southern Afghanistan and western Pakistan. Mitochondrial genetic distance values between these lineages are greater than between the two recognised species *T*. *roborowskii* and *T*. *przewalskii* (S3 Table). These lineages thus should be taxonomically revised including specimens from the type locality of *T*. *microlepis* in Iran. A taxonomic revision should also include *T*. *sistanensis*, which is morphologically closest to *T*. *microlepis* [79]. These two species are distinguished by the number of dorsal scales around mid-body (85–110 in *T*. *microlepis* and 145–165 in *T*. *sistanensis*) [79]. Each lineage of *T*. *microlepis* from our study exhibits characters belonging to the two species (examined specimens from the MVZ collection by TJP; lineage I/A, 98–188 dorsal scales around mid-body and lineage II/B, 87–165). Since *T*. *sistanensis* was described based on morphological data alone and the lack of additional distinguishing characters known, further studies are needed to investigate these taxa, considering the type localities of the two species.

A second case is the non-monophyly of *T*. *keyserlingii*, with respect to *T*. *mesriensis*, and the presence of five distinct lineages within it. The lineages recovered within *T*. *keyserlingii*, including that of *T*. *mesriensis*, diverged in the Pliocene and Pleistocene, and are geographically structured within Iran and neighbouring countries. For both *T*. *microlepis* and *T*. *keyserlingii* we hypothesise that the tectonic consequences of the northward motion of the Arabian plate and collision with the Eurasian landmass, resulted in intense geological instability and uplift of mountain ranges, as well as associated climatic changes prevailing in the Miocene to Pleistocene, most likely prompted these diversifications [80]. A positive association between intraspecific diversity, exhibited as genetic structure and/or structured lineage distribution, and habitat discontinuity is expected [81]. We therefore call for thorough systematic revisions of these two species, with broad geographic sampling of specimens to account for the large amount of spatial and genetic diversity, with the plausible finding of additional lineages.

### Origin of the Arabian population of *T*. *keyserlingii*

The UAE specimens of *T*. *keyserlingii* cluster together in lineage IV/E with one specimen from southern Iran, near the coastal city of Bandar Abbas. Low levels of genetic divergence between the coastal southern Iran sample and the Arabian population leads us to hypothesise that Arabia was colonised from southern Iran relatively recently, during the Pleistocene. Connections between Arabia and Iran have been recorded for several reptile taxa, including *Acanthodactylus* [82], *Mesalina* [83,84], *Hemidactylus* [85,86], and *Pristurus* [87]. As has been hypothesised in the case of *Pseudocerastes persicus* [88], colonization could have occurred by transmarine dispersal across the Strait of Hormuz, or directly by land dispersal during the last glaciations when sea levels were low [89]; another plausible scenario is human-mediated dispersal, although being a secretive, sand-dwelling, nocturnal reptile makes this hypothesis less likely. Additionally, recent divergence is indicated by extremely low genetic variability within the Arabian population of *T*. *keyserlingii*. Several UAE specimens differentiate by a single mutation in the mitochondrial data indicating rapid recent expansion across the UAE.

### Population structure of *T*. *keyserlingii* in the UAE

Genetic structure analysis with DAPC shows the UAE *T*. *keyserlingii* population lacking any internal geographic structure, hence representing a single population. In fact, this pattern is in agreement with the genetic variation measures (percentage of polymorphic loci and polymorphic sites) that highlight the extremely low genetic diversity within the studied system. Recently, a study that also used a ddRAD approach estimated within-population π values for more than 80 lizard populations ranging from 2.14x10^-4^ to 3.99x10^-3^ [90]. Our estimated π value within *T*. *keyserlingii* in the UAE is much lower than the lowest estimated value in the aforementioned study. Accordingly, Tajima D test of neutrality returned a negative value of -2.233. Negative values are indicative of recent selective sweeps or, more possibly in our case, population expansions after a recent bottleneck or a founder event, which is congruent with our divergence time estimation of Arabian colonization during the late Pleistocene.

Results from DAPC and STRUCTURE seem contradictory at first glance: the former identified no genetic structure within *T*. *keyserlingii* in the UAE, while the latter detected three sub-populations. The DAPC analysis does not require a model of admixture and describes the relationship between the population clusters, optimizing the variance between groups while minimizing variance within groups. In this sense, it is expected to perform better when organisms exhibit continuous spatial population structure [91]. On the other hand, the algorithm implemented in STRUCTURE assumes a specific evolutionary model, under which a more or less equal number of individuals are randomly sampled from K populations, each of which is characterised by a set of allele frequencies at each locus. It is further assumed that within populations, loci are at HW and linkage equilibrium, while the effect of genetic drift and lack of gene-flow will lead to linkage disequilibrium and departures from Hardy-Weinberg proportions, when there is population structure. The STRUCTURE algorithm uses this signal to detect the “true” number of populations [62].

**Fig 6:**
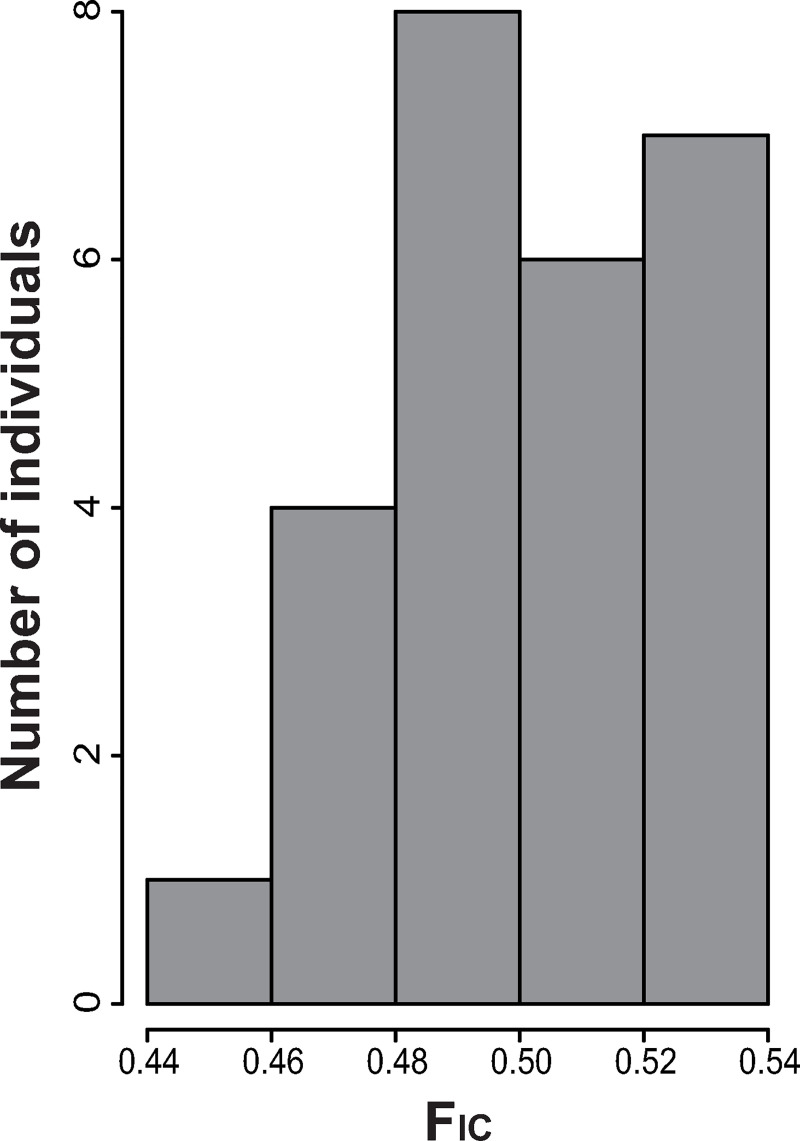
Inbreeding frequency distribution. Calculated for the 26 individuals of *T*. *keyserlingii* from the UAE.

Any major violation of the former assumptions can lead to severe under- or overestimation of the number of K. For example, representing some populations with only very few individuals is one possible violation and simulation studies have shown that this may affect the estimated K [63]. Following Wang (2017) [63], our analyses were run under a scheme aiming to correct for unbalanced sampling violations, without altering the outcome of K = 3. However, there is strong evidence that this clustering does not represent “true” population structure of *T*. *keyserlingii* in the UAE. Firstly, where several values of K give similar estimates of LnP(X/K), as here for K = 1 and K = 3, the smallest value is often “correct” [92]. Secondly, the split to three clusters most probably reflects departures from the STRUCTURE model that are detected as HW or linkage disequilibrium within the assumed populations. Inbreeding, specifically, can lead to a weak statistical signal for K>1, even in the absence of population structure [93]. The mean inbreeding coefficient (F_IC_) per individual in our sample showed high values of F_IC_>0.45 (Fig 6). This indicates an almost 50% probability that individuals with common alleles at any locus show this genetic similarity by descent from genetically related parents. This would be a serious violation of the random mating assumed by the STRUCTURE model, forcing the algorithm to group together the most closely related individuals and in a distinct cluster all non-related (or less related) ones. Upon inspection of the aligned consensus sequences, we found that each of the two local clusters defined by STRUCTURE included individuals that shared an extremely high number of common alleles in approximately 94% of the total polymorphic sites, or 348–350 of 370 total uSNPs. The effect of inbreeding also explains the unreasonably high divergence estimated among the three clusters; classical F statistics consider independent populations and are not suitable for populations connected with extensive gene-flow [94].

Conclusively the bulk of evidence suggests that there is no true structure within our samples and that *T*. *keyserlingii* specimens from the UAE belong to a single population. This population shows a low level of genetic diversity, an excess of homozygotes, and high levels of inbreeding, all consistent with a recent founder event. Non-random mating leading to high values of F_IC_ can be induced by bottlenecks or founder events, small population size and habitat fragmentation in natural populations (e.g., [95,96]), but it can also be the result of breeding programs on captive animals [97]. In any case, increased inbreeding may lead to the fixation of deleterious alleles and is associated with higher mortality, lower fertility and even extinction [98,99].

### Conservation implications for *T*. *keyserlingii* in the UAE

Effects of anthropogenic activities on natural environments and biodiversity by habitat destruction and fragmentation are considered major drivers for the decline and categorization of *Teratoscincus keyserlingii* as Critically Endangered in the UAE. Its decline is related to the environmental characteristics of small patches of natural ecosystems: they comprise lower environmental heterogeneity, they are occupied by small populations that are more vulnerable to local extinction, they have limited immigration rate, and their edges represent degraded habitat for many species [15].

Biodiversity conservation starts with knowledge of genetic diversity within and among populations. A high level of genetic diversity in a population is thought to increase the probability of surviving a disastrous event or extinction [12]. Thus, in order to implement effective management policies, i.e., to prioritise protection status or to determine re-introduction or translocation options, it is imperative to know the genetic structure of focal populations. Our genomic conclusions of a single population of *T*. *keyserlingii* in the UAE, with no internal structure, rather simplify possible immediate conservation strategies (i.e., not accounting for the loss of genetic diversity). This genetic inference therefore has significant implications for conservation management.

**Fig 7:**
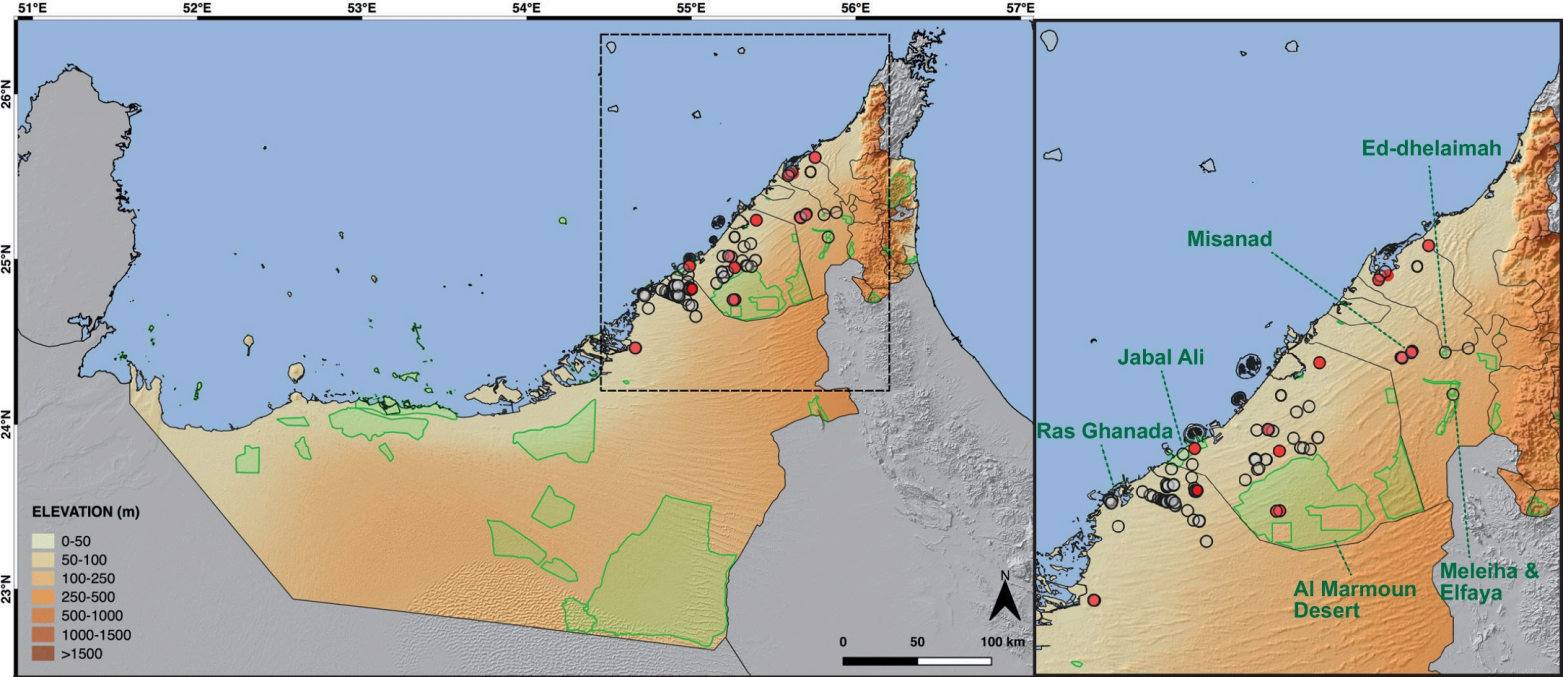
Distribution of *T*. *keyserlingii* in the UAE within the current terrestrial protected areas. Terrestrial protected areas are indicated in green (see details in Burriel-Carranza et al., 2019) [28]. Circles indicate *T*. *keyserlingii* specimens used in this study (red) and past known localities (blank). Credits: OpenStreetMap contributors, SRTM. Shapefile of the terrestrial protected areas of the UAE is provided in S1 Appendix.

The UAE currently includes 42 protected areas, covering 16.6% of the terrestrial area of the country [28], albeit most of the protected range is located at the southern edge of the Abu Dhabi Emirate (Fig 7). Burriel-Carranza et al. (2019) [28] calculated the percentage of *T*. *keyserlingii* distribution included within these protected areas, showing that it did not reach the strict Aichi biodiversity conservation target of 17% and only slightly surpassed the less restrictive 12% target. Their study indicated an immediate problem regarding habitat loss of *T*. *keyserlingii* and its low presence in protected areas. Currently, three protected areas (Jabal Ali, Al Marmoun Desert, and Misanad) incorporate areas of *T*. *keyserlingii* distribution in the UAE, three include past localities of the species (Ed-dhelaimah, Meleiha and Elfaya, and Ras Ghanada), and the rest are either too small and scattered, or located beyond the natural habitat of the species (Fig 7). Unfortunately, most protected areas covering currently observed specimens of *T*. *keyserlingii* are small, containing minimal overlap with the species’ overall distribution in the UAE, are located near developing areas, or are too scattered to maintain natural environments with habitat connectivity. Species distribution modelling presented in Burriel-Carranza et al. (2019) [28] indicated that the distribution of *T*. *keyserlingii* may be extended to southern Dubai and northern Abu Dhabi Emirates. As protection of the environment is being recognised as an essential component of the natural development policies in the UAE, one achievable proposition is expansion of current protected areas (e.g., northern expansion of Al Marmoun Desert reserve in Dubai), or establishment of new areas with suitable habitats for *T*. *keyserlingii* (e.g., in northern Abu Dhabi Emirate, west of Al Marmoun Desert reserve). These spatial zones are of critical importance to the natural preservation of the species in the UAE. In this regard, improved land management and natural habitat restoration in disturbed areas are also plausible efforts to improve the quality, connectivity, and permeability for future dispersal of different species, as well as preserving the biological diversity and stability of the ecosystem [100].

In the UAE there are currently two breeding centres for endangered Arabian wildlife, including *T*. *keyserlingii*–in Abu Dhabi and Sharjah Emirates. The goal of many *ex-situ* conservation agendas, such as captive breeding or re-introduction programs, is retention of a high percentage of genetic diversity from wild populations, until its potential re-introduction to natural habitat to ensure the long-term viability of the founder populations [101–103] (see also [104] for further references and discussion). However, increased inbreeding originating from a small number of stock parents may lead to fixation of deleterious alleles and is associated with higher mortality, lower fertility and even extinction [98,99]. Additionally, the strategy of releasing inbred animals locally may result in extreme local homogeneity. Both cases will result in fixation of alleles throughout the UAE population, or at least locally. Coupled with small home ranges and limited dispersal distances per generation, re-introduction should thus be taken into consideration for future release of inbred animals and a more efficient re-location strategy. Thus, e*x-situ* conservation programs have to be carefully integrated within *in-situ* programs, so that captive populations can constitute an insurance against the loss of natural populations and a source for re-introduction projects [15]. Yet, when the decline of species has reached catastrophic dimensions, as is predicted for *T*. *keyserlingii*, captive breeding and other *ex-situ* conservation programs should not be considered alone as efficient conservation measures without additional *in-situ* measures [105], and must not become an excuse to avoid dealing with the preservation of natural habitats.

Several decades of habitat destruction and fragmentation have led to the gradual disappearance of the Arabian population of *T*. *keyserlingii*. As shown in Fig 2, between 1984 and 2020, massive development in the coastal areas of the Emirates of Abu Dhabi, Dubai, Sharjah, Umm Al Quwain and Ras al Khaimah have exterminated many localities where *T*. *keyserlingii* had been previously reported (Fig 7) and have artificially fragmented the species’ distribution range. The genomic inferences in this study enable the re-location of specimens from current areas of occupancy, from destroyed habitats or breeding centres, to areas where the probability of future persistence is predicted to be higher. Setting up of a solid and continuous education and awareness campaign will help to ensure constant, but increasingly necessary, actions for the preservation of *T*. *keyserlingii* in Arabia. In addition, the protection of *T*. *keyserlingii* will indisputably simultaneously promote protection of other co-occurring species. The primary habitat of *T*. *keyserlingii* consists of sand sheets, low undulating sand dunes and sandy plains with relatively dense vegetation dominated by the grasses *Pennisetum divisum* and *Panicum turgidum* [26]. These vegetated coastal sandy areas of the UAE represent a unique ecosystem in urgent need of protection, that is the natural habitat of a large community of geckos, as well as of many other animals both vertebrate and invertebrate [26,28].

## Supporting information

S1 Fig The mPTP results of *Teratoscincus*. Results inferred from the concatenated mitochondrial dataset. Sample codes correlate to specimens in S1 Table and in Figs 2 and 5.
(DOCX)

S2 Fig Bayesian values *vs*. Number of clusters as resulted from DAPC analysis. Analysis based on 26 individuals of *T*. *keyserlingii* from the UAE used for the ddRAD analyses. The Bayesian values do not improve as the clusters increase from 1 to 10 implying K = 1.
(DOCX)

S3 Fig Bayesian genetic clustering of the 26 individuals of *T*. *keyserlingii* from the UAE using the program STRUCTURE. (A) The optimal clustering was identified using Evanno’s method (ΔK plotted against K) at K = 3. (B) The division or runs is also given (similar clustering in any subset of the total 15 runs per K).
(DOCX)

S1 Table Information on the *Teratoscincus* specimens included in the analyses and related GenBank accession numbers. Localities of specimens from the UAE are indicated next to the country’s name and in Fig 2. Specimen code abbreviations: [CAS] California Academy of Sciences, San Francisco, USA; [CN] Institute of Evolutionary Biology, Barcelona, Spain; [JFBM] J. F. Bell Museum of Natural History, University of Minnesota, USA; [KU] University of Kansas Biodiversity Institution, Lawrence, USA; [MVZ Herp] Museum of Vertebrate Zoology, University of California, Berkeley, USA; [TG] Tony Gamble collection; [ZMMU] Zoological Museum of Moscow State University, Moscow, Russia. (*) *Teratoscincus keyserlingii* specimens used for the ddRADseq analyses (n = 26).
(DOCX)

S2 Table Data on the gene fragments used in this study. Information including the primers used, with their orientation, sequences, references, and PCR conditions.
(DOCX)

S3 Table Pairwise uncorrected mitochondrial sequence divergence (*p*-distance) among and within *Teratoscincus*. Values derived from the mitochondrial gene fragments of *COI* (below diagonal) and *ND2* (above diagonal), and within each taxon or lineage (in bold; *COI*/*ND2*).
(DOCX)

S1 Appendix Shapefile of the terrestrial protected areas of the UAE. (ZIP)

S2 Appendix ddRAD data generated in this study. (RAR)
