# Supplementary material for: The demise of a wonder: Evolutionary history and conservation assessments of the Wonder Gecko Teratoscincus keyserlingii (Gekkota, Sphaerodactylidae) in Arabia
Source: PLoS One. 2021 Jan 7;16(1):e0244150. doi: 10.1371/journal.pone.0244150 (PMC7790289; doi:10.1371/journal.pone.0244150)
Supplement: S1 Fig — Results inferred from the concatenated mitochondrial dataset. Sample codes correlate to specimens in S1 Table and in Figs 2 and 5. (DOCX) [file pone.0244150.s001.docx]

**S1 Figure.** The mPTP results of *Teratoscincus* inferred from the concatenated mitochondrial dataset. Sample codes correlate to specimens in S1 Table and in Figs 2 and 5.

**
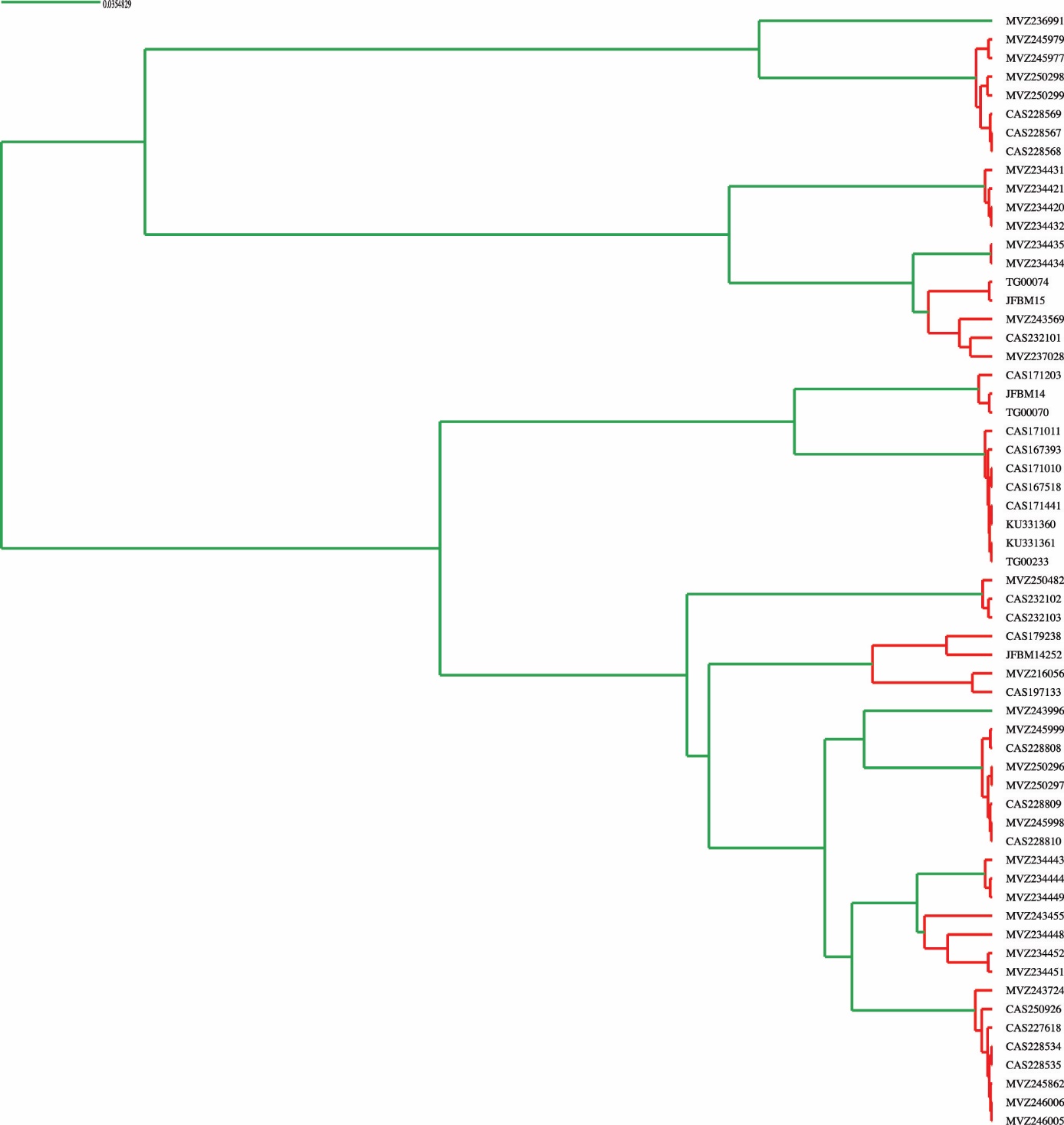
**
