# Supplementary material for: The demise of a wonder: Evolutionary history and conservation assessments of the Wonder Gecko Teratoscincus keyserlingii (Gekkota, Sphaerodactylidae) in Arabia
Source: PLoS One. 2021 Jan 7;16(1):e0244150. doi: 10.1371/journal.pone.0244150 (PMC7790289; doi:10.1371/journal.pone.0244150)

**S2 Figure.** Bayesian values vs. Number of clusters as resulted from DAPC analysis of the 26 individuals of *T. keyserlingii* from the UAE used for the ddRAD analyses. The Bayesian values do not improve as the clusters increase from 1 to 10 implying K=1.


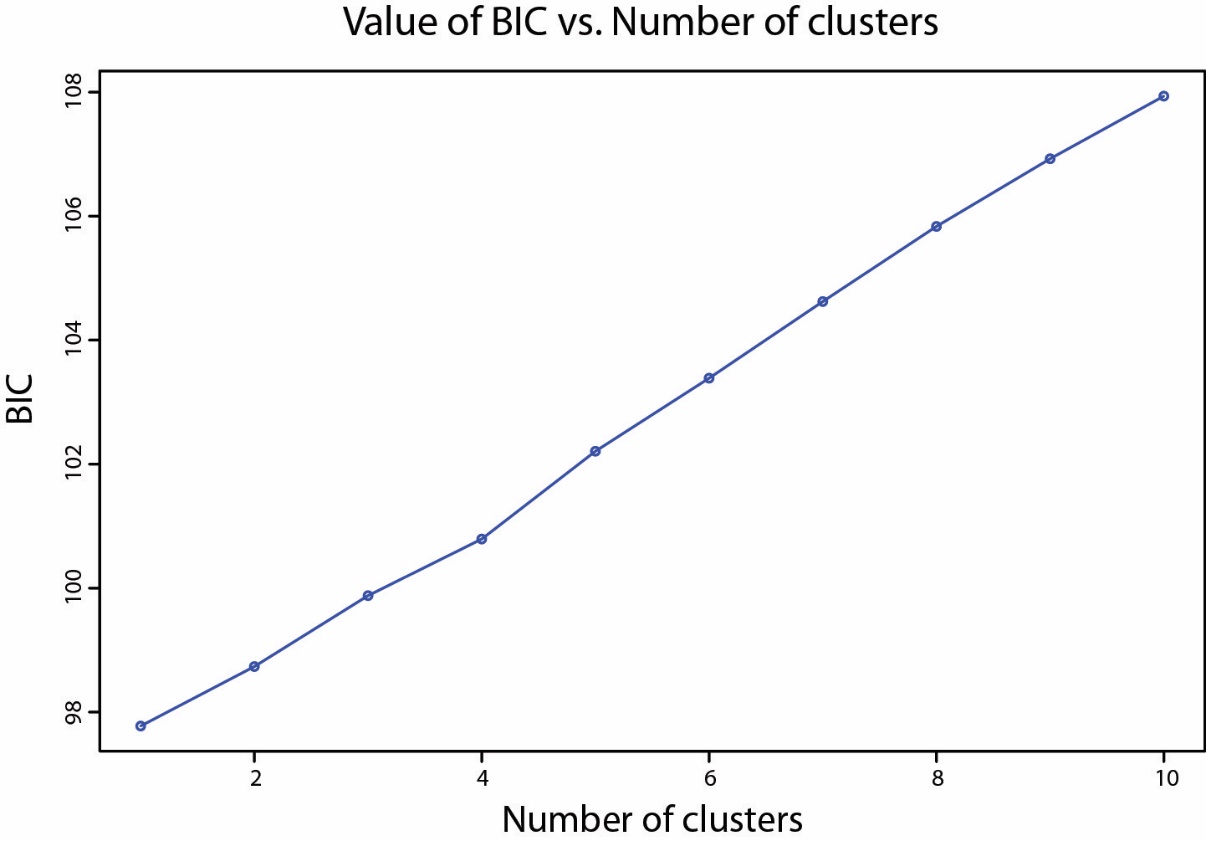

Supplement: S2 Fig — Analysis based on 26 individuals of T. keyserlingii from the UAE used for the ddRAD analyses. The Bayesian values do not improve as the clusters increase from 1 to 10 implying K = 1. (DOCX) [file pone.0244150.s002.docx]
