# Supplementary material for: The demise of a wonder: Evolutionary history and conservation assessments of the Wonder Gecko Teratoscincus keyserlingii (Gekkota, Sphaerodactylidae) in Arabia
Source: PLoS One. 2021 Jan 7;16(1):e0244150. doi: 10.1371/journal.pone.0244150 (PMC7790289; doi:10.1371/journal.pone.0244150)

**S3 Figure.** Bayesian genetic clustering of the 26 individuals of *T. keyserlingii* from the UAE using the program STRUCTURE. (A) The optimal clustering was identified using Evanno’s method (ΔK plotted against K) at K=3. (B) The division or runs is also given (similar clustering in any subset of the total 15 runs per K).

(A)


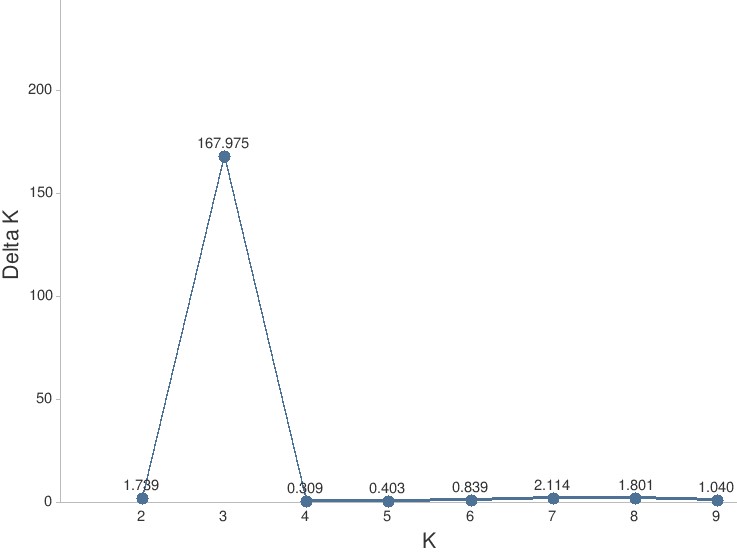


(B)


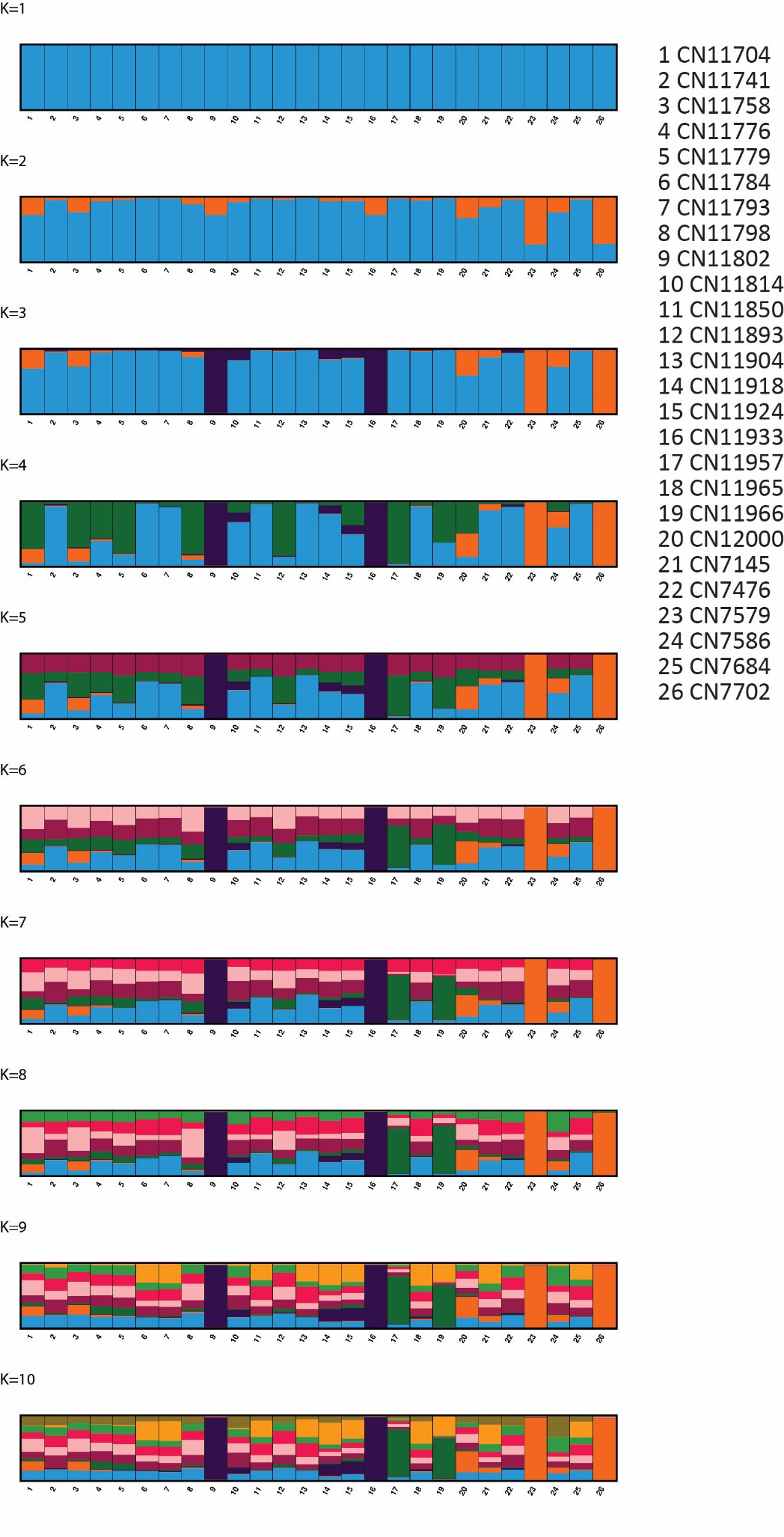

Supplement: S3 Fig — (A) The optimal clustering was identified using Evanno’s method (ΔK plotted against K) at K = 3. (B) The division or runs is also given (similar clustering in any subset of the total 15 runs per K). (DOCX) [file pone.0244150.s003.docx]
