# Supplementary material for: The demise of a wonder: Evolutionary history and conservation assessments of the Wonder Gecko Teratoscincus keyserlingii (Gekkota, Sphaerodactylidae) in Arabia
Source: PLoS One. 2021 Jan 7;16(1):e0244150. doi: 10.1371/journal.pone.0244150 (PMC7790289; doi:10.1371/journal.pone.0244150)
Supplement: S1 Table — Localities of specimens from the UAE are indicated next to the country’s name and in Fig 2. Specimen code abbreviations: [CAS] California Academy of Sciences, San Francisco, USA; [CN] Institute of Evolutionary Biology, Barcelona, Spain; [JFBM] J. F. Bell Museum of Natural History, University of Minnesota, USA; [KU] University of Kansas Biodiversity Institution, Lawrence, USA; [MVZ Herp] Museum of Vertebrate Zoology, University of California, Berkeley, USA; [TG] Tony Gamble collection; [ZMMU] Zoological Museum of Moscow State University, Moscow, Russia. (*) Teratoscincus keyserlingii specimens used for the ddRADseq analyses (n = 26). (DOCX) [file pone.0244150.s004.docx]

**S1 Table. Information on the *Teratoscincus* specimens included in the analyses and related GenBank accession numbers.** Localities of specimens from the UAE are indicated next to the country’s name and in Fig 2. Specimen code abbreviations: [CAS] California Academy of Sciences, San Francisco, USA; [CN] Institute of Evolutionary Biology, Barcelona, Spain; [JFBM] J. F. Bell Museum of Natural History, University of Minnesota, USA; [KU] University of Kansas Biodiversity Institution, Lawrence, USA; [MVZ Herp] Museum of Vertebrate Zoology, University of California, Berkeley, USA; [TG] Tony Gamble collection; [ZMMU] Zoological Museum of Moscow State University, Moscow, Russia. (*) *Teratoscincus keyserlingii* specimens used for the ddRADseq analyses (n=26).

| **Specimen Code** | **Species** | **Lineage** | **Country** | **Lat** | **Lon** | ***COI*** | ***ND2*** | ***MC1R*** | ***RAG1*** |
| --- | --- | --- | --- | --- | --- | --- | --- | --- | --- |
| CAS228567 | *Teratoscincus bedriagai* |  | Iran | 35.951 | 56.091 | MT977213 | MT977395 | MT977306 | MT977254 |
| CAS228568 | *Teratoscincus bedriagai* |  | Iran | 35.951 | 56.091 | MT977214 | MT977396 | - | MT977255 |
| CAS228569 | *Teratoscincus bedriagai* |  | Iran | 35.951 | 56.091 | MT977215 | MT977397 | MT977311 | MT977258 |
| MVZ Herp 236991 | *Teratoscincus bedriagai* |  | Afghanistan | 31.383 | 65.892 | MT977220 | MT977402 | MT977313 | MT977261 |
| MVZ Herp 245977 | *Teratoscincus bedriagai* |  | Iran | 35.951 | 56.091 | MT977218 | MT977398 | MT977307 | MT977256 |
| MVZ Herp 245979 | *Teratoscincus bedriagai* |  | Iran | 35.951 | 56.091 | MT977216 | MT977399 | MT977308 | MT977259 |
| MVZ Herp 250298 | *Teratoscincus bedriagai* |  | Iran | 35.951 | 56.091 | MT977217 | MT977400 | MT977309 | MT977257 |
| MVZ Herp 250299 | *Teratoscincus bedriagai* |  | Iran | 35.951 | 56.091 | MT977219 | MT977401 | MT977310 | MT977260 |
| ZMMURAN039a | *Teratoscincus bedriagai* |  | Iran |  |  | MF573788 | - | - | - |
| ZMMURAN1912 | *Teratoscincus bedriagai* |  | Iran |  |  | MF573789 | - | - | - |
| ZMMURAN1913 | *Teratoscincus bedriagai* |  | Iran |  |  | MF573787 | - | - | - |
| ZMMURAN1914 | *Teratoscincus bedriagai* |  | Iran |  |  | MF573790 | - | - | - |
| CAS232102 | *Teratoscincus* *keyserlingii* | A/I | Pakistan | 29.470 | 65.981 | MT977200 | MT977332 | MT977281 | MT977235 |
| CAS232103 | *Teratoscincus* *keyserlingii* | A/I | Pakistan | 29.470 | 65.981 | MT977201 | MT977333 | MT977280 | MT977236 |
| MVZ Herp 250482 | *Teratoscincus* *keyserlingii* | A/I | Pakistan | 29.470 | 65.981 | MT977202 | MT977334 | MT977291 | MT977243 |
| MVZ Herp 245998 | *Teratoscincus* *keyserlingii* | B/II | Iran | 32.492 | 57.018 | MT977189 | MT977391 | MT977288 | MT977252 |
| MVZ Herp 245999 | *Teratoscincus* *keyserlingii* | B/II | Iran | 32.492 | 57.018 | MT977190 | MT977388 | MT977290 | MT977246 |
| MVZ Herp 250296 | *Teratoscincus keyserlingii* | B/II | Iran | 32.492 | 57.018 | MT977191 | MT977392 | MT977303 | MT977253 |
| MVZ Herp 250297 | *Teratoscincus keyserlingii* | B/II | Iran | 32.492 | 57.018 | MT977192 | MT977393 | MT977289 | MT977247 |
| CAS228808 | *Teratoscincus* *keyserlingii* | B/II | Iran | 32.492 | 57.018 | MT977186 | MT977387 | MT977283 | MT977250 |
| CAS228809 | *Teratoscincus* *keyserlingii* | B/II | Iran | 32.492 | 57.018 | MT977187 | MT977389 | MT977284 | - |
| CAS228810 | *Teratoscincus* *keyserlingii* | B/II | Iran | 32.492 | 57.018 | MT977188 | MT977390 | MT977301 | MT977251 |
| ZMMURAN1931a | *Teratoscincus keyserlingii* | C | Iran |  |  | MF573792 | - | - | - |
| ZMMURAN1931b | *Teratoscincus keyserlingii* | C | Iran |  |  | MF573791 | - | - | - |
| ZMMURAN242 | *Teratoscincus keyserlingii* | C | Iran | 34.710 | 58.800 | MF573793 | - | - | - |
| MVZ Herp 234443 | *Teratoscincus keyserlingii* | D/III | Iran | 29.487 | 60.797 | MT977194 | MT977381 | MT977302 | MT977230 |
| MVZ Herp 234444 | *Teratoscincus keyserlingii* | D/III | Iran | 29.487 | 60.797 | MT977195 | MT977382 | MT977285 | MT977229 |
| MVZ Herp 234448 | *Teratoscincus keyserlingii* | D/III | Iran | 29.475 | 60.790 | MT977197 | MT977386 | MT977286 | MT977231 |
| MVZ Herp 234449 | *Teratoscincus keyserlingii* | D/III | Iran | 29.475 | 60.790 | MT977196 | MT977383 | MT977287 | MT977228 |
| MVZ Herp 234451 | *Teratoscincus keyserlingii* | D/III | Iran | 27.253 | 60.409 | MT977198 | MT977384 | MT977304 | MT977238 |
| MVZ Herp 234452 | *Teratoscincus keyserlingii* | D/III | Iran | 27.253 | 60.409 | MT977199 | MT977385 | MT977299 | MT977239 |
| MVZ Herp 243455 | *Teratoscincus keyserlingii* | D/III | Iran | 30.446 | 57.833 | AY753545 | AY753545 | - | - |
| CAS228534 | *Teratoscincus* *keyserlingii* | E/IV | UAE_9 | 24.957 | 54.989 | MT977141 | MT977336 | MT977293 | MT977233 |
| CAS228535 | *Teratoscincus* *keyserlingii* | E/IV | UAE_9 | 24.957 | 54.989 | MT977142 | MT977337 | MT977294 | MT977234 |
| CAS227618 | *Teratoscincus* *keyserlingii* | E/IV | UAE_3 | 25.506 | 55.590 | MT977140 | MT977335 | MT977292 | MT977232 |
| CAS250926 | *Teratoscincus* *keyserlingii* | E/IV | UAE_8 | 24.949 | 55.266 | MT977176 | MT977341 | MT977295 | MT977237 |
| CN11704* | *Teratoscincus* *keyserlingii* | E/IV | UAE_12 | 24.820 | 54.991 | MT977150 | MT977348 | - | - |
| CN11726 | *Teratoscincus* *keyserlingii* | E/IV | UAE_12 | 24.820 | 54.991 | MT977151 | MT977349 | - | - |
| CN11729 | *Teratoscincus* *keyserlingii* | E/IV | UAE_12 | 24.820 | 54.991 | MT977152 | MT977379 | - | - |
| CN11740 | *Teratoscincus* *keyserlingii* | E/IV | UAE_5 | 25.253 | 55.663 | MT977153 | MT977373 | - | - |
| CN11741* | *Teratoscincus* *keyserlingii* | E/IV | UAE_4 | 25.273 | 55.700 | MT977154 | MT977374 | - | - |
| CN11758* | *Teratoscincus* *keyserlingii* | E/IV | UAE_12 | 24.820 | 54.991 | MT977155 | MT977350 | - | - |
| CN11776* | *Teratoscincus* *keyserlingii* | E/IV | UAE_7 | 25.019 | 55.230 | MT977156 | MT977351 | - | - |
| CN11779* | *Teratoscincus* *keyserlingii* | E/IV | UAE_11 | 24.754 | 55.267 | MT977157 | MT977352 | - | - |
| CN11784* | *Teratoscincus* *keyserlingii* | E/IV | UAE_5 | 25.251 | 55.667 | MT977158 | MT977353 | - | - |
| CN11793* | *Teratoscincus* *keyserlingii* | E/IV | UAE_5 | 25.253 | 55.667 | MT977159 | MT977375 | - | - |
| CN11798* | *Teratoscincus* *keyserlingii* | E/IV | UAE_6 | 25.236 | 55.397 | MT977160 | MT977354 | - | - |
| CN11802* | *Teratoscincus* *keyserlingii* | E/IV | UAE_7 | 25.019 | 55.227 | MT977181 | MT977355 | - | - |
| CN11814* | *Teratoscincus* *keyserlingii* | E/IV | UAE_7 | 25.019 | 55.227 | MT977182 | MT977356 | - | - |
| CN11826 | *Teratoscincus* *keyserlingii* | E/IV | UAE_4 | 25.273 | 55.700 | MT977161 | MT977377 | - | - |
| CN11850* | *Teratoscincus* *keyserlingii* | E/IV | UAE_10 | 24.753 | 55.255 | MT977177 | MT977357 | - | - |
| CN11858 | *Teratoscincus* *keyserlingii* | E/IV | UAE_6 | 25.236 | 55.397 | MT977162 | MT977358 | - | - |
| CN11862 | *Teratoscincus* *keyserlingii* | E/IV | UAE_6 | 25.236 | 55.397 | MT977183 | MT977359 | - | - |
| CN11863 | *Teratoscincus* *keyserlingii* | E/IV | UAE_6 | 25.236 | 55.397 | MT977163 | MT977360 | - | - |
| CN11893* | *Teratoscincus* *keyserlingii* | E/IV | UAE_6 | 25.236 | 55.397 | MT977164 | MT977361 | - | - |
| CN11904* | *Teratoscincus* *keyserlingii* | E/IV | UAE_6 | 25.236 | 55.397 | MT977165 | MT977376 | - | - |
| CN11918* | *Teratoscincus* *keyserlingii* | E/IV | UAE_7 | 25.019 | 55.230 | MT977184 | MT977362 | - | - |
| CN11924* | *Teratoscincus* *keyserlingii* | E/IV | UAE_7 | 25.019 | 55.227 | MT977178 | MT977363 | - | - |
| CN11933* | *Teratoscincus* *keyserlingii* | E/IV | UAE_7 | 25.019 | 55.230 | MT977185 | MT977364 | - | - |
| CN11957* | *Teratoscincus* *keyserlingii* | E/IV | UAE_6 | 25.236 | 55.397 | MT977166 | MT977365 | - | - |
| CN11965* | *Teratoscincus* *keyserlingii* | E/IV | UAE_6 | 25.236 | 55.397 | MT977167 | MT977366 | - | - |
| CN11966* | *Teratoscincus* *keyserlingii* | E/IV | UAE_6 | 25.236 | 55.397 | MT977168 | MT977367 | - | - |
| CN12000* | *Teratoscincus* *keyserlingii* | E/IV | UAE_6 | 25.236 | 55.397 | MT977179 | MT977368 | - | - |
| CN12011 | *Teratoscincus* *keyserlingii* | E/IV | UAE_12 | 24.820 | 54.991 | MT977169 | MT977369 | - | - |
| CN12025 | *Teratoscincus* *keyserlingii* | E/IV | UAE_4 | 25.269 | 55.697 | MT977170 | MT977370 | - | - |
| CN12053 | *Teratoscincus* *keyserlingii* | E/IV | UAE_12 | 24.820 | 54.991 | MT977171 | MT977378 | - | - |
| CN7145* | *Teratoscincus* *keyserlingii* | E/IV | UAE_13 | 24.464 | 54.658 | MT977175 | MT977342 | - | - |
| CN7476* | *Teratoscincus* *keyserlingii* | E/IV | UAE_1 | 25.617 | 55.754 | MT977143 | MT977371 | - | - |
| CN7574 | *Teratoscincus* *keyserlingii* | E/IV | UAE_12 | 24.820 | 54.991 | MT977144 | MT977343 | - | - |
| CN7579* | *Teratoscincus* *keyserlingii* | E/IV | UAE_12 | 24.820 | 54.991 | MT977145 | MT977344 | - | - |
| CN7586* | *Teratoscincus* *keyserlingii* | E/IV | UAE_12 | 24.820 | 54.991 | MT977146 | MT977345 | - | - |
| CN7684* | *Teratoscincus* *keyserlingii* | E/IV | UAE_12 | 24.820 | 54.991 | MT977147 | MT977346 | - | - |
| CN7702* | *Teratoscincus* *keyserlingii* | E/IV | UAE_12 | 24.820 | 54.991 | MT977148 | MT977347 | - | - |
| CN7791 | *Teratoscincus* *keyserlingii* | E/IV | UAE_1 | 25.616 | 55.753 | MT977149 | MT977372 | - | - |
| MVZ Herp 243724 | *Teratoscincus* *keyserlingii* | E/IV | Iran | 27.289 | 56.474 | MT977180 | MT977380 | MT977300 | MT977244 |
| MVZ Herp 245862 | *Teratoscincus* *keyserlingii* | E/IV | UAE_2 | 25.520 | 55.620 | MT977172 | MT977338 | MT977296 | MT977240 |
| MVZ Herp 246005 | *Teratoscincus* *keyserlingii* | E/IV | UAE_9 | 24.957 | 54.989 | MT977173 | MT977339 | MT977297 | MT977241 |
| MVZ Herp 246006 | *Teratoscincus* *keyserlingii* | E/IV | UAE_9 | 24.957 | 54.989 | MT977174 | MT977340 | MT977298 | MT977242 |
| MVZ Herp 243996 | *Teratoscincus mesriensis* |  | Iran | 31.625 | 54.398 | MT977193 | MT977394 | MT977305 | MT977245 |
| ZMMURAN2536 | *Teratoscincus mesriensis* |  | Iran |  |  | MF573794 | - | - | - |
| ZMMURAN2537 | *Teratoscincus mesriensis* |  | Iran |  |  | MF573795 | - | - | - |
| CAS232101 | *Teratoscincus microlepis* | A/I | Pakistan | 29.470 | 65.981 | MT977203 | MT977403 | MT977314 | MT977262 |
| MVZ Herp 234434 | *Teratoscincus microlepis* | A/I | Iran | 25.270 | 60.755 | MT977206 | MT977406 | MT977315 | MT977264 |
| MVZ Herp 234435 | *Teratoscincus microlepis* | A/I | Iran | 25.270 | 60.755 | MT977207 | MT977407 | MT977316 | MT977265 |
| MVZ Herp 237028 | *Teratoscincus microlepis* | A/I | Afghanistan | 31.383 | 65.892 | MT977204 | MT977404 | - | - |
| MVZ Herp 243569 | *Teratoscincus microlepis* | A/I | Iran | 31.119 | 61.602 | MT977205 | MT977405 | MT977312 | MT977263 |
| TG00074 | *Teratoscincus microlepis* | A/I | Pakistan |  |  | MT977208 | JX041451 | MT977317 | EF534800 |
| JFBM15 | *Teratoscincus microlepis* | A/I | Pakistan |  |  | - | KU158023 | - | KU157921 |
| MVZ Herp 234420 | *Teratoscincus microlepis* | B/II | Iran | 27.254 | 60.409 | MT977209 | MT977408 | MT977318 | MT977266 |
| MVZ Herp 234421 | *Teratoscincus microlepis* | B/II | Iran | 27.254 | 60.409 | MT977212 | MT977409 | MT977321 | MT977268 |
| MVZ Herp 234431 | *Teratoscincus microlepis* | B/II | Iran | 26.963 | 60.146 | MT977210 | MT977411 | MT977319 | MT977267 |
| MVZ Herp 234432 | *Teratoscincus microlepis* | B/II | Iran | 26.963 | 60.146 | MT977211 | MT977410 | MT977320 | MT977269 |
| ZMMUR11736 | *Teratoscincus microlepis* | B/II | Iran |  |  | MF573800 | - | - | - |
| ZMMURAN1189 | *Teratoscincus microlepis* | B/II | Iran |  |  | MF573798 | - | - | - |
| CAS167393 | *Teratoscincus przewalskii* |  | China | 40.090 | 94.678 | MT977129 | MT977322 | MT977270 | MT977221 |
| CAS167518 | *Teratoscincus przewalskii* |  | China | 39.023 | 88.155 | MT977130 | MT977323 | MT977271 | MT977222 |
| CAS171011 | *Teratoscincus przewalskii* |  | China | 42.844 | 93.824 | MT977131 | MT977324 | MT977272 | MT977223 |
| CAS171441 | *Teratoscincus przewalskii* |  | China | 45.554 | 85.069 | MT977132 | MT977325 | MT977273 | MT977225 |
| KU331360 | *Teratoscincus przewalskii* |  | Mongolia | 43.250 | 98.990 | MT977133 | MT977326 | MT977275 | MT977226 |
| KU331361 | *Teratoscincus przewalskii* |  | Mongolia | 43.250 | 98.990 | MT977134 | MT977327 | MT977274 | MT977227 |
| CAS171010 | *Teratoscincus przewalskii* |  | China | 42.840 | 93.824 | - | TPU71326 | - | AY662624 |
| TG00233 | *Teratoscincus przewalskii* |  | China |  |  | - | JX041452 | - | HQ426335 |
| ZMMUR120441 | *Teratoscincus przewalskii* |  | Mongolia |  |  | MF573797 | - | - | - |
| ZMMUR131221 | *Teratoscincus przewalskii* |  | China |  |  | MF573796 | - | - | - |
| ZMMUR131222 | *Teratoscincus przewalskii* |  | China |  |  | MF573799 | - | - | - |
| ZMMUR131223 | *Teratoscincus przewalskii* |  | China |  |  | MF573801 | - | - | - |
| CAS171203 | *Teratoscincus roborowskii* |  | China | 42.867 | 90.375 | MT977135 | MT977328 | MT977276 | MT977224 |
| TG00070 | *Teratoscincus roborowskii* |  | China |  |  | MT977136 | JX041453 | MT977277 | EF534799 |
| JFBM14 | *Teratoscincus roborowskii* |  |  |  |  | - | KU158024 | - | KU157922 |
| ZMMURAN1297a | *Teratoscincus roborowskii* |  | China |  |  | MF573802 | - | - | - |
| ZMMURAN1993 | *Teratoscincus roborowskii* |  | China |  |  | MF573803 | - | - | - |
| ZMMURAN702 | *Teratoscincus roborowskii* |  | China |  |  | MF573804 | - | - | - |
| ZMMUR110691 | *Teratoscincus rustamowi* |  | Uzbekistan |  |  | MF573805 | - | - | - |
| CAS179238 | *Teratoscincus scincus* |  | Turkmenistan | 38.581 | 63.171 | MT977137 | MT977329 | MT977278 | MT977248 |
| CAS197133 | *Teratoscincus scincus* |  | China | 43.969 | 80.844 | MT977139 | MT977331 | MT977279 | MT977249 |
| JFBM14252 | *Teratoscincus scincus* |  | Turkmenistan |  |  | MT977138 | MT977330 | MT977282 | EF534801 |
| ZMMUR10064 | *Teratoscincus scincus* |  | Uzbekistan |  |  | MF573810 | - | - | - |
| ZMMUR108651 | *Teratoscincus scincus* |  | Uzbekistan |  |  | MF573809 | - | - | - |
| ZMMUR114201 | *Teratoscincus scincus* |  | Uzbekistan |  |  | MF573807 | - | - | - |
| ZMMUR114221 | *Teratoscincus scincus* |  | Uzbekistan |  |  | MF573806 | - | - | - |
| ZMMUR5817 | *Teratoscincus scincus* |  | Tajikistan |  |  | MF573808 | - | - | - |
| MVZ Herp 216056 | *Teratoscincus scincus* |  | Kazakhstan | 43.150 | 76.570 | - | AF114251 | - | - |
| ZMMURL32 | *Teratoscincus scincus* |  | Uzbekistan |  |  | MF573811 | - | - | - |
