# Supplementary material for: The demise of a wonder: Evolutionary history and conservation assessments of the Wonder Gecko Teratoscincus keyserlingii (Gekkota, Sphaerodactylidae) in Arabia
Source: PLoS One. 2021 Jan 7;16(1):e0244150. doi: 10.1371/journal.pone.0244150 (PMC7790289; doi:10.1371/journal.pone.0244150)
Supplement: S2 Table — Information including the primers used, with their orientation, sequences, references, and PCR conditions. (DOCX) [file pone.0244150.s005.docx]

**S2 Table.** **Data on the gene fragments used in this study.** Information including the primers used, with their orientation, sequences, references, and PCR conditions.

| **Gene** | **Length** | **Primer** | **Sequence (5’-3’)** | **Reference** | **PCR Conditions** |
| --- | --- | --- | --- | --- | --- |
| *COI* | 651 | RepCOI_F | F: TNTTMTCAACNAACCACAAAGA | Nagy et al. (2012) | 94°C (3’), [94°C (40’’), 50°C (30’’), 72°C (1’)] x35, 72°C (5’) |
|  |  | RepCOI_R | R: ACTTCTGGRTGKCCAAARAATCA |  |  |
| *ND2* | 546 | ND2_Ts_L4437b | F: AAGCTTTCGGGCCCATACC | Macey et al. (1997) | 95°C (2’), [95°C (35’’), 50°C (35’’), 72°C (150’’)] x33, 72°C (5’) |
|  |  | ND2­_Ts_r102 | R: CAYCCYATGTGGGCRATTG | Greenbaum et al. (2007) |  |
| *MC1R* | 663 | MC1R-F | F: AGGCNGCCATYGTCAAGAACCGGAACC | Pinho et al. (2009) | 94°C (5’), [94°C (30’’), 58°C (45’’), 72°C (80’’)] x35, 72°C (5’) |
|  |  | MC1R-R | R: CTCCGRAAGGCRTAAATGATGGGGTCCAC |  |  |
| *RAG1* | 1008 | RAG1_R13 | F: TCTGAATGGAAATTCAAGCTGTT | Groth & Barrowclough (1999) | 95°C (2’), [95°C (35’’), 60°C (1’), 72°C (150’’)] x35, 72°C (5’) |
|  |  | RAG1_R18 | R: GATGCTGCCTCGGTCGGCCACCTTT |  |  |

**References**

Greenbaum E, Bauer AM, Jackman TR, Vences M, Glaw F. A phylogeny of the enigmatic Madagascan geckos of the genus *Uroplatus* (Sauria: Gekkonidae). Zootaxa. 2207; 1493, 41–51.

Groth JG, Barrowclough GF. Basal Divergences in Birds and the Phylogenetic Utility of the Nuclear RAG-1 Gene. Mol Phylogenet Evol. 1999; 12, 115–123.

Macey JR, Larson A, Ananjeva NB, Fang Z, Papenfuss TJ. Two novel gene orders and the role of light-strand replication in rearrangement of the vertebrate mitochondrial genome. Mol Biol Evol. 1997; 14, 91–104

Nagy ZT, Sonet G, Glaw F, Vences M. First large-scale DNA barcoding assessment of reptiles in the biodiversity hotspot of Madagascar, based on newly designed COI primers. PLoS One. 2012; 7, e34506.

Pinho C, Rocha S, Carvalho BM, Lopes S, Mourao S, Vallinoto M, Brunes TO, Haddad CFB, Goncalves H, Sequeira F, Ferrand N. New primers for the amplification and sequencing of nuclear loci in a taxonomically wide set of reptiles and amphibians. Conserv. Genet. Resour. 2009; 2(S1), 181–185.
