# Supplementary material for: The demise of a wonder: Evolutionary history and conservation assessments of the Wonder Gecko Teratoscincus keyserlingii (Gekkota, Sphaerodactylidae) in Arabia
Source: PLoS One. 2021 Jan 7;16(1):e0244150. doi: 10.1371/journal.pone.0244150 (PMC7790289; doi:10.1371/journal.pone.0244150)
Supplement: S3 Table — Values derived from the mitochondrial gene fragments of COI (below diagonal) and ND2 (above diagonal), and within each taxon or lineage (in bold; COI/ND2). (DOCX) [file pone.0244150.s006.docx]

**S3 Table.** **Pairwise uncorrected mitochondrial sequence divergence (*p*-distance) among and within *Teratoscincus*.** Values derived from the mitochondrial gene fragments of *COI* (below diagonal) and *ND2* (above diagonal), and within each taxon (in bold; *COI*/*ND2*).

| **Taxon** | | 1 | 2 | 3 | 4 | 5 | 6 | 7 | 8 | 9 | 10 | 11 | 12 | 13 |
| --- | --- | --- | --- | --- | --- | --- | --- | --- | --- | --- | --- | --- | --- | --- |
| 1. | *T. bedriagai* | **1.4/2.5** | 14.5 | 16.5 | 17.3 | 15.8 | 16.1 | - | 16.5 | 17 | - | 16.8 | 15.3 | 17 |
| 2. | *T. microlepis* A | 16.5 | **1.5/1.7** | 8.5 | 16.5 | 17.5 | 15.8 | - | 16.8 | 16.6 | - | 16.5 | 17 | 17 |
| 3. | *T. microlepis* B | 16.2 | 8.3 | **2.6/0.2** | 19.6 | 20.3 | 17.5 | - | 18.2 | 19 | - | 17.8 | 18.9 | 18.9 |
| 4. | *T. roborowskii* | 16.7 | 18.7 | 18 | **0.8/0.7** | 7.5 | 12.7 | - | 13.3 | 12.5 | - | 13.4 | 13.1 | 13.2 |
| 5. | *T. przewalskii* | 15.6 | 17.1 | 16.8 | 6.4 | **0.0/0.1** | 14.7 | - | 14.1 | 15.9 | - | 16.3 | 14.8 | 15 |
| 6. | *T. scincus* | 16.7 | 15.7 | 15.9 | 13.2 | 11.5 | **2.3/4.25** | - | 9.2 | 8.3 | - | 9.4 | 9.8 | 8.7 |
| 7. | *T. rustamowi* | 16.8 | 14.2 | 14.6 | 14.1 | 12 | 6.3 | **NA** | - | - | - | - | - | - |
| 8. | *T. keyserlingii* A | 16.8 | 15.8 | 15.8 | 15.2 | 13.8 | 8.5 | 7.5 | **0.2/0.4** | 9.9 | - | 10 | 9.4 | 9.3 |
| 9. | *T. keyserlingii* B | 16.4 | 15 | 14.7 | 13.5 | 11.8 | 7.3 | 6.6 | 8.2 | **0.0/0.3** | - | 5.6 | 6.5 | 5.1 |
| 10. | *T. keyserlingii* C | 16 | 14.5 | 14.7 | 13.5 | 12.3 | 7.3 | 6.5 | 7.8 | 3.9 | **0.0/-** | - | - | - |
| 11. | *T. mesriensis* | 16.9 | 15.3 | 15.3 | 13.3 | 12.5 | 8 | 7.4 | 9.5 | 5.2 | 3.4 | **2.2/NC** | 7.8 | 6.5 |
| 12. | *T. keyserlingii* D | 17 | 15.5 | 15.9 | 13.6 | 12.7 | 8.8 | 8.3 | 9.3 | 8.2 | 4.7 | 5.5 | **2.8/2.3** | 5.1 |
| 13. | *T. keyserlingii* E | 16.5 | 15.3 | 15.3 | 14.1 | 12.8 | 8.3 | 8 | 8.9 | 4.2 | 3.4 | 4.7 | 5.4 | **0.1/0.2** |
